# Using multiple modalities to confirm diagnosis in patients with suspected peroxisome biogenesis disorders

**DOI:** 10.1016/j.ymgme.2025.109080

**Published:** 2025-03-11

**Authors:** Anthony C.T. Cheung, Erminia Di Pietro, Catherine Argyriou, Eric Bareke, Yasmin D’Souza, Ratna Dua Puri, P. Muhammed Shabeer, Rebecca Ganetzky, Amy Goldstein, Adeline Vanderver, Shruthi Mohan, Jacek Majewski, Christine Yergeau, Nancy Braverman

**Affiliations:** a Division of Medical Genetics, Department of Specialized Medicine, McGill University Health Center, Montreal, Quebec, Canada; b Department of Human Genetics, McGill University, Montreal, Quebec, Canada; c Institute of Medical Genetics and Genomics, Sir Ganga Ram Hospital, Delhi, India; d Mitochondrial Medicine Frontier Program, Division of Human Genetics, Department of Pediatrics, Children’s Hospital of Philadelphia, Philadelphia, PA, USA; e Division of Neurology, Children’s Hospital of Philadelphia, Philadelphia, PA, USA; f Department of Pathology, Duke University School of Medicine, Durham, NC, USA; g Departments of Human Genetics and Pediatrics, McGill University, Montreal, Quebec, Canada; h Research Institute of the McGill University Health Centre, Montreal, Quebec, Canada; i Department of Pediatrics, Perelman School of Medicine, University of Pennsylvania, Philadelphia, PA, USA; j Department of Neurology, Perlman School of Medicine, University of Pennsylvania, Philadelphia, PA, USA

**Keywords:** Peroxisome biogenesis disorder, Zellweger spectrum disorder, PEX1, RNA sequencing, Intronic variant, Splice-site variant

## Abstract

Zellweger spectrum disorder (ZSD) results from biallelic variants in any one of 13 *PEX* genes involved in peroxisome biogenesis and function. The majority of ZSD cases result from pathogenic variants in *PEX1*. Here, we present 3 patients with suspected *PEX1*-related ZSD and non-diagnostic whole exome sequencing and describe the use of multiple modalities to ascertain their diagnosis. We confirmed peroxisomal dysfunction in the patients by demonstrating abnormal peroxisome metabolite levels in blood and peroxisome import dysfunction in patient fibroblasts. RNA studies including RNA-seq and RT-PCR, followed by Sanger sequencing showed leaky splice variants including an intron 13 variant causing exon 14 skipping (Patient 1), an intron 22 variant causing intron 22 retention (Patient 2), and a synonymous splice-site variant causing exon 16 skipping (Patient 3). All three patients had very low amounts of canonical PEX1 transcripts on RNA-seq, as well as residual but reduced PEX1 protein levels on immunoblotting, which likely explains their non-severe ZSD phenotype. This study suggests that a multi-modality approach combining biochemical testing, functional assays in fibroblasts and molecular investigations including sequencing of non-coding regions and RNA analysis may aid in diagnosis of patients with suspected PBD-ZSD and inconclusive WES.

## 1. Introduction

Peroxisome biogenesis disorders (PBD) are a heterogeneous group of conditions resulting from defects in any one of 14 *PEX* genes involved in peroxisome assembly and function. Deleterious variants in any of 13 of these genes lead to Zellweger spectrum disorders, the largest subgroup of PBDs that comprises a spectrum of clinical phenotypes and severity. Approximately 60–70 % of ZSD cases in North America result from pathogenic variants in *PEX1* [1–3]. Patients with the most severe phenotype present with congenital malformations including polymicrogyria, cortical renal cysts, chondrodysplasia punctata (limited to hips and knees), and generally do not survive beyond the first year of life. Intermediate and mild forms of ZSD have a wide range of clinical manifestations that commonly include amelogenesis imperfecta, progressive retinal degeneration, and sensorineural hearing loss. These patients are also at risk of having developmental delay, liver dysfunction, adrenal insufficiency, nephrolithiasis, and leukodystrophy [4–6].

The diagnosis of PBDs is made via biochemical and molecular testing. Analysis of multiple peroxisome metabolites in plasma and/or patient fibroblasts, as well as localization of peroxisomal enzymes in cultured fibroblasts have traditionally been used to determine peroxisomal dysfunction. With the advent of next generation sequencing (NGS), definitive diagnosis of PBD is made when biallelic *PEX* gene variants are identified, although *PEX6* pseudo-dominant [7] and *PEX14* de novo dominant alleles [8] have been recently described. Two null alleles in a *PEX* gene are generally associated with a severe phenotype, whereas the presence of at least one hypomorphic allele typically confers either an intermediate or mild phenotype [9,10]. While gene panels and whole exome sequencing (WES) are primarily used to ascertain the genetic cause of patients suspected to have PBD, these conventional NGS methods fail to identify causative biallelic variants in some patients. Recently, a patient with features of mild ZSD and only one pathogenic *PEX1* variant on WES was reported to harbor a deep intronic *PEX1* variant identified *in trans* via RNA sequencing (RNA-seq), highlighting the need to sequence non-coding regions in patients with inconclusive WES [11].

We present here 3 patients with suspected *PEX1*-related ZSD but non-diagnostic WES results. We describe the use of multiple diagnostic modalities, including biochemical testing, functional assays on patient fibroblasts, RNA-seq, and reverse transcription-polymerase chain reaction (RT-PCR) to confirm peroxisomal dysfunction and underlying genetic defect(s) of ZSD.

## 2. Methods

### 2.1. Clinical data

Patients were recruited through our institutional research ethics board-approved Longitudinal Natural History Study of Patients with Peroxisome Biogenesis Disorders (ClinicalTrials.gov Identifier: NCT01668186). Consent for research and publication of clinical information was obtained from all patients and their families. Patient medical information, MRI imaging and fibroblast cultures were collected through the Natural History Study. Peroxisome function tests and gene sequencing were performed in clinically certified laboratories, except where indicated.

### 2.2. In silico analyses

Splicing effects of intronic variants and splice site variants were analyzed using Human Splice Finder and SpliceAI [12,13].

### 2.3. Molecular biology

Primary fibroblast cell lines, passage 2–20 were cultured at 37 °C, 7 % CO_2_ in DMEM with 10 % FBS. Genotypes of cell lines used to compare PEX1 protein levels were *PEX1* c.[2528G > A];[2528G > A]; PEX1 p.[G843D];[G843D], PEX1 c.[2528G > A];[382C > T]; p.[G843D];[0], *PEX1* c.[2097_2098insT]; [2097_2098insT]; PEX1 p.[0];[0], and non-disease control. Genotypes for the 3 patients studied in this report are found in results section. Cells were grown in flasks or on glass coverslips and processed at full confluence for RNA and immunoblot, and approximately 70 % confluence for immunofluorescent assays.

### 2.4. RNA-seq

RNA was extracted from fibroblasts suspended in Trizol (concentration 1 × 10^7 cells/900 uL) using RNeasy Plus Mini kit (QIAGEN, Hilden, Germany). The RNA integrity was assessed with an Agilent 2100 Bioanalyzer (Agilent Technologies). The corresponding RNA libraries were constructed following the New England Biolabs RNA stranded Library protocol (Illumina Library QC). The RNA libraries were sequenced using NovaSeq 6000 PE100 system (Illumina). The Centre d’expertise et de service Génome Québec performed RNA extraction and sequencing. The Canadian Center for Computational Genomics (C3G) performed sequence quality control, annotation to reference genomes and quantification of gene counts using the GenPipes pipeline [14]. The RNA-seq StringTie workflow was followed from GenPipes version 3.1.5. (https://bitbucket.org/mugqic/genpipes/src/master/pipelines/rnaseq/). Adaptor sequences and low-quality scoring bases from sequenced reads (Phred score < 30) were trimmed using Trimmomatic version 0.36 [15]. The resulting reads from Human were aligned to *Homo sapiens* GRCh37 (release 75), using Spliced Transcripts Alignment to a Reference (STAR) software, version 2.7.8a [16]. Read count were obtained using HTseq version 0.6.1p1 [17].

### 2.5. RT-PCR

Total RNA was purified using RNeasy mini kit and RT-PCR performed with OneStep RT-PCR kit (Qiagen) with the following parameters: Reverse transcription: At 50 °C for 30 min, initial PCR activation: 95 °C for 15 min followed by 30 cycles of 94 °C for 30 s, 55 °C for 30 s, and 72 °C for 1 min and a final extension at 72 °C for 10 min.

PEX1 cDNA fragments amplified and primer pairs used:

For patient 1: exon 13 (forward) 5’-CCATGGGAAGTTTGGTTGCA-3′ and exon 15 (reverse) 5’-TCTGCCTAACTTCATGTAACCC-3′; exon 12 (forward) 5’-TGGCTTTCTCAGAGGCAGTG-3′ and exon 15 (reverse) 5’-CTTGGCAGGTAACTGGATAGT-3′.

For patient 2: exon 22 (forward) 5’-TCTGCACCAAGCTCCATGAC-3′ and exon 24 reverse 5’-ACAGTGAAGAAGCTGCTAAGCATAA-3′; exon 18 (forward) 5’-CTGCAAAGCCCTGCATTCTT-3′ and exon 23 (reverse) 5’-TGACTGCACTTGGTCACACA-3′.

For Patient 3: exon 15 (forward) 5’-TTAACAACATTGGACTTCCAAAAGG-3′ and exon 17 (reverse) 5’-ATATCCCGAACAGCTTGTTCACT-3′. cDNA fragments were cloned using TA cloning kit (Topo pCR2.1 or ThermoFisher pJet2.1). PCR products were Sanger sequenced (Genome Quebec, Montreal) and variants were detected using Sequencher software V4.9 (Gene Codes Corp.).

### 2.6. Immunoblotting

Fibroblasts lysates were prepared in IGEPAL lysis buffer. Lysates (20 μg) were separated on 7.5 % polyacrylamide gels and transferred to nitrocellulose membranes. Membranes were blocked and hybridized in 5 % milk with primary antibodies: 1:1000 rabbit anti-human PEX1 (Proteintech (Rosemont, IL) 13669–1-AP), 1:2000 rabbit anti-human PEX5, PEX6 (G. Dodt, University of Tübingen) 1:17000 rabbit anti-human β-tubulin (Abcam (Cambridge, MA) ab6046), followed by HRP-conjugated secondary antibodies, and visualized by ECL using an Amersham 600 platform. Band quantification (densitometry) was done using ImageJ (NIH).

### 2.7. Indirect immunofluorescence

Cells were fixed, permeabilized and incubated with antiserum as reported [18]. Primary antibodies were rabbit anti-PEX5 1:300 (G. Dodt, University of Tübingen) and anti-SKL 1:300 (S. Gould, Johns Hopkins University), rabbit anti-catalase 1:300 (AOXRE 24316, Burlingame, California), mouse anti-PMP70 1:150 (Sigma SAB4200181, St. Louis, Missouri). Secondary antibodies were goat anti-rabbit 488 1:400 (Invitrogen A-11008, Waltham, Massachusetts) and goat anti-mouse 594 1:300 (InvitrogenA-11005, Waltham, Massachusetts). Images were visualized using a Leica DMI600 microscope with a DFC345FX camera and LASX software (Leica, Richmond Hill, ON, Canada). Scoring of protein localization was performed as reported [19].

### 2.8. Peroxisome metabolite analysis

Patients 1 and 2 had testing done from blood in clinical laboratories. Patient 3 had C26:0 lysophosphatidylcholine (lyso-PC) and plasmalogen analysis done from fibroblast lysate by liquid chromatography-tandem mass spectrometry (LC-MS/MS) as reported [19].

### 2.9. Statistical analysis

Results are expressed as mean ± standard deviation (SD). Comparisons of multiple measures per patient cell line to non-disease control were performed using 2-way ANOVA with Bonferroni post-test. Comparisons of a single measure per patient cell line compared to non-disease control were performed using 1-way ANOVA with Neuman-Keuls post-test. Statistical significance was set based on *P* value: *P < 0.05, **P < 0.01, ***P < 0.001, ****P < 0.0001. A minimum sample size of 3 was used for all comparisons. Statistical analysis was performed using GraphPad Prism 10.0 software.

## 3. Results

### 3.1. Case reports

Clinical features are summarized in Table 1 and peroxisome metabolite levels are listed in Table 2. Patient 1 was born at 42 weeks to non-consanguineous parents after a normal pregnancy conceived by artificial insemination. He was noted to have delays in gross and fine motor, language, and cognitive functions by age 3 years. He had progressive motor dysfunction starting in his late teens characterized by generalized dystonia affecting the face, neck, and all extremities, rigidity, spasticity, bradykinesia and cerebellar dysfunction including ataxia and dysarthria. His dystonia responded partially to Levodopa-Carbidopa with improvement in tone, tremors and dysarthria. However, increased fatigue led to the discontinuation of the medication. He completed a high school education at age 21 years and his cognitive function remained relatively stable. High frequency sensorineural hearing loss (SNHL) was diagnosed in childhood. Ophthalmological exam at 19 years was normal. Brain MRI at 26 years showed cruciform signal abnormality in the pons, T2 hyperintensity in the middle cerebellar peduncles and marked pontocerebellar atrophy. Trio WES (patient and parents) at 31 years revealed compound heterozygous variants in *PEX1*: c.2097dupT (p. Ile700TyrfsX42) and c.2227–11 T > A (IVS 13–11 T > A), inherited *in trans* and classified respectively as pathogenic and variant of uncertain significance (VUS). In silico analysis of the intronic variant using Human Splice Finder predicted the generation of a new donor splice site (+17.49 %), while SpliceAI did not predict acceptor loss (Δ score 0.02). Trio whole genome sequencing done as follow-up investigation was unrevealing. Measurement of peroxisome metabolites at 31 years showed mild elevations in very long chain fatty acids (VLCFAs) and mild reduction in plasmalogens (see Table 2).

Patient 2 was born at 37 weeks to non-consanguineous parents after an uncomplicated pregnancy. She was noted to have hypotonia, feeding difficulties and dysmorphic features including large anterior fontanelle, frontal bossing, and epicanthal folds at birth. She had global developmental delay, meeting her 5-month-old milestones by age 3 years. She had episodes of motor regression during acute illness. Feeding difficulties including gastroparesis and gastroesophageal reflux disease persisted throughout life and required gastrostomy tube insertion at age 1 year. She had SNHL and cortical visual impairment, although her retinas were noted to be normal. She developed infantile myoclonic epilepsy responsive to Clobazam at age 7 months, and episodes of multifocal tremors that had no electrographic correlate on electroencephalography. She also had temperature dysregulation, intermittent hyperhidrosis and cold discolored extremities, suggestive of autonomic nervous system involvement. She had serial brain MRIs that showed ventriculomegaly and required a ventriculoperitoneal shunt at age 1.5 years. Liver and renal function tests and abdominal imaging were all normal. Extensive metabolic investigations including CSF neurotransmitters, mitochondrial electron transport chain enzymes, and mitochondrial DNA content and sequencing on muscle tissue were normal. Testing of peroxisome metabolites at age 1 year showed elevated VLCFAs and mild decrease in erythrocyte plasmalogens (Table 2). However, trio WES at 2.5 years revealed only one paternally-inherited pathogenic variant in *PEX1*: c.2916delA (p. Gly973Alafs*16). The patient died at age 3 years from a presumed infection.

Patient 3 was born at term to non-consanguineous parents. Antenatal history and delivery were uneventful, and development was normal. Newborn hearing screening via otoacoustic emission showed absence of response bilaterally. At age 4.5 years, brainstem auditory evoked response testing showed bilateral moderate to severe SNHL, which was corrected with hearing aids. At age 7 years, she was found to have a learning disorder as well as inattention and hyperactivity, but had otherwise normal intelligence. She has normal vision, teeth enamel and neurological exam. Physical exam is remarkable for pitting in all nails, a finding associated with a mild form of ZSD [9,20]. Electroretinogram and optical coherence tomography at age 6.5 years, as well as brain MRI at 4.5 years were normal. Peroxisome metabolites were not obtained from Patient 3. Trio WES at 6 years revealed homozygous synonymous variants *PEX1* c.2718G > A (p. Lys906(=)) located at the last nucleotide of exon 16 which were segregated in heterozygous state in the parents and interpreted as VUS. Human Splice Finder predicts this variant to cause a broken splice donor (−10.88 %), while SpliceAI predicts a donor gain (Δ score 0.73).

Given the inconclusive WES results in all patients and mild biochemical abnormalities in Patients 1 and 2, we further evaluated peroxisome functions in primary fibroblasts in each patient as discussed below.

### 3.2. Peroxisome metabolites

These were performed in fibroblasts from patient 3 by LC-MS/MS. C26:0 lyso-PC levels were elevated and plasmalogen levels were reduced (Table 2).

### 3.3. Peroxisome import studies

**Fig. 1.**
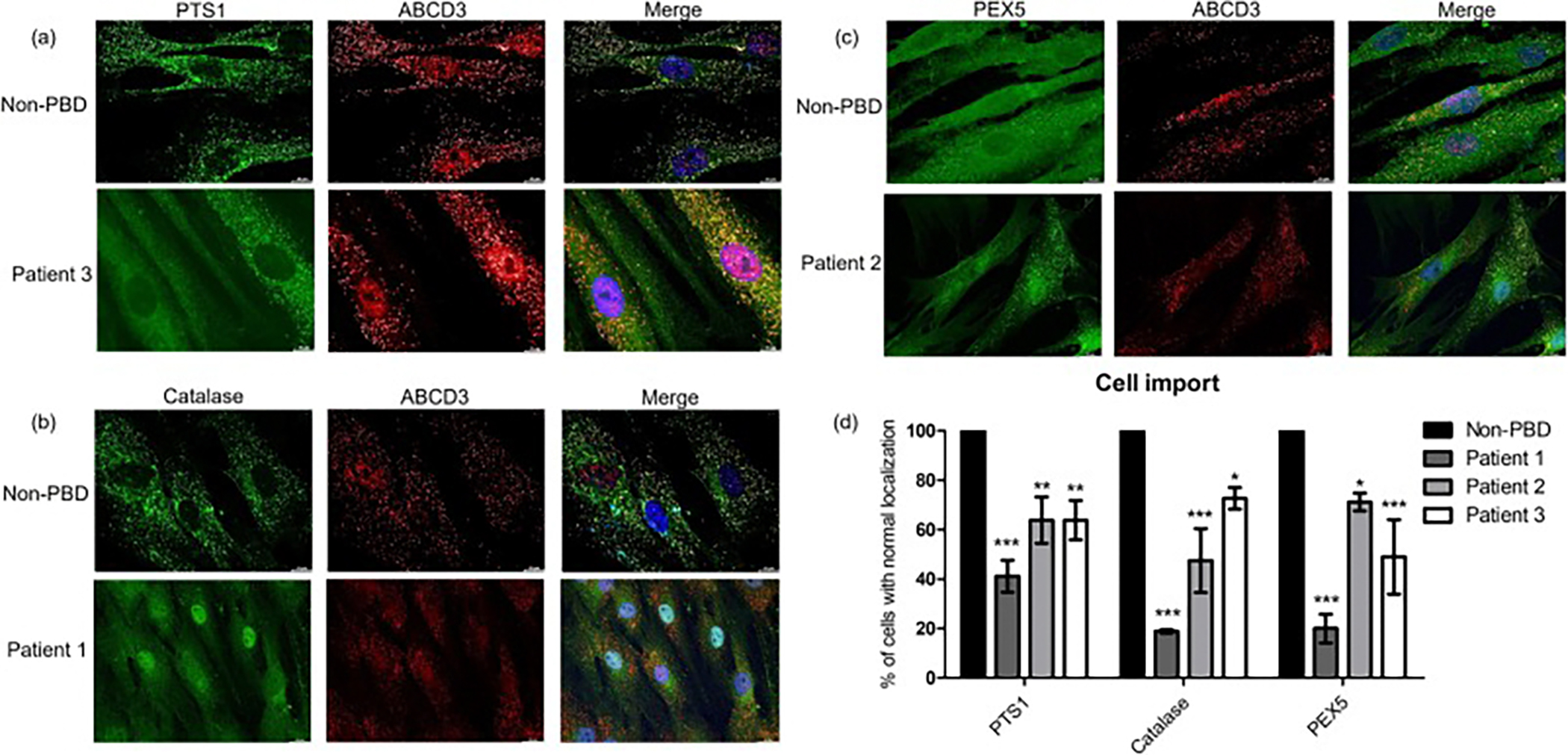
Markers of peroxisome function show abnormal localization in patient primary fibroblasts. Import function of patient fibroblasts was studied using indirect immunofluorescent microscopy to visualize localization of endogenous (a), PTS1 proteins and (b), catalase which normally show localization to the peroxisome matrix, and (c), PEX5, which normally localizes to the cytosol. Note “mosaicism” present in (a) where two of four cells are importing PTS1 proteins, and (c) where one of three cells has cytosolic PEX5 localization. ABCD3 was used as a peroxisome membrane marker to validate co-localization of proteins to the peroxisome (merge image). One representative image per patient cell line and for each marker is shown. Histogram (d), shows percent of cells with proper PTS1, catalase, and PEX5 localization for each cell line (mean ± SD). Proportion of cells with normal localization of each protein were calculated by scoring at least 100 cells per experiment. *N* = 3; 2-way ANOVA with Bonferroni post-test.; * = *p* ≤ 0.05, ** = *p* ≤ 0.01, *** = *p* ≤ 0.001 compared to non-PBD control. (PTS1 = peroxisome targeting signal 1, or C-terminal serine, lysine, leucine motif detected by anti-SKL antiserum; ABCD3 = ATP binding cassette subfamily D member 3).

To assess peroxisomal function, we evaluated the localization of PTS1, catalase, and PEX5 markers in primary fibroblast cell lines from all three patients (Fig. 1). We show one representative image for each patient cell line and marker (Fig. 1a, patient 3 and PTS1 enzyme import; 1b, patient 1 and catalase import; 1c, patient 2 and PEX5 localization; 1d, percent of cells with normal localization of each marker). ABCD3, an integral peroxisome membrane protein, was used to confirm co-localization of the marker with peroxisomes.

Most matrix enzymes are imported into peroxisomes via their endogenous peroxisome targeting signal 1 motif (PTS1). Thus, this is a robust marker for matrix enzyme import; peroxisomal (punctate) or cytosolic PTS1 localization is indicative of importing or non-importing cells, respectively (Fig. 1a). PTS1 localization was abnormal in 60 % of cells from Patient 1 (40 % normal localization), and 36 % of cells from Patients 2 and 3 (64 % normal localization), on average (Fig. 1d). Catalase is the most sensitive marker of PTS1 protein import because it has a non-consensus signal and therefore depends on relatively high integrity of the peroxisome import system (Fig. 1b) [21]. The localization of catalase was abnormal in 80 % of cells from Patient 1, 52 % of cells from Patient 2, and 27 % of cells from Patient 3 (Fig. 1d). We also examined the localization of the peroxisome PTS1 receptor PEX5. The PEX1-PEX6 complex functions to recycle PEX5 from the peroxisome membrane to enable additional rounds of import [22]. When this export process is impaired, PEX5 becomes stuck at the peroxisome membrane, exhibiting punctate localization instead of the normal cytosolic distribution (Fig. 1c). PEX5 localization was abnormal in 80 % of cells from Patient 1, 29 % of cells from Patient 2, and 51 % of cells from Patient 3 (Fig. 1d). Taken together, these observations support impaired peroxisomal functions in cell lines from all 3 patients.

Peroxisomal “mosaicism” a term used to describe functional peroxisomes in cell culture adjacent to cells lacking functional peroxisomes and is observed in patients with milder forms of ZSD [23]. This was observed in all patient cell lines for the makers used and can be seen in Fig. 1a for PTS1 import and Fig. 1c for PEX5 recycling.

### 3.4. Transcript analysis of PEX1 variants

#### 3.4.1. PEX1 transcript levels

**Fig. 2.**
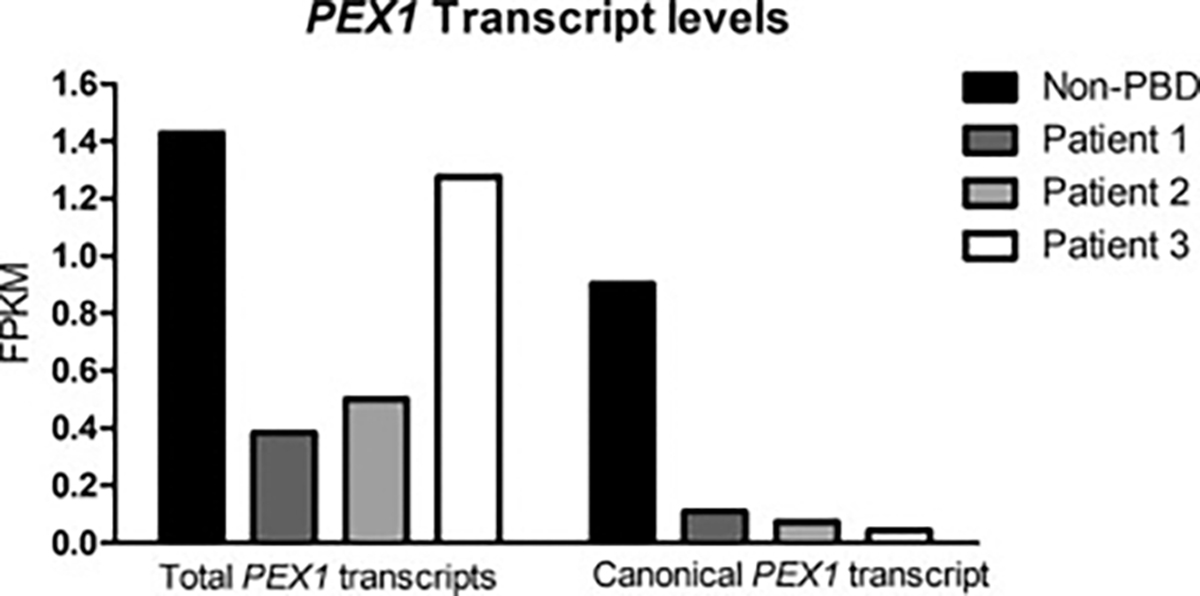
PEX1 transcript levels in patient fibroblasts. Total *PEX1* transcripts (including all *PEX1* isoforms as curated by Ensembl) as well as the canonical PEX1 transcript (ENST00000248633) were present at reduced amounts in patient fibroblasts compared to non-PBD controls. Transcript abundance is represented in fragments per kilobase per million mapped fragments (FPKM).

RNA-seq analysis was performed on fibroblast RNA from all three patient cell lines and a non-PBD control cell line (Fig. 2). *PEX1* transcript levels from all 12 identified *PEX1* isoforms are provided in Supplemental Table 1. Total *PEX1* transcript in cells from patient 1 was 26.8 % of non-PBD, 35.1 % and 89.3 % for patient 2 and 3 respectively (Fig. 2 left). Fig. 2, right, shows amounts of canonical *PEX1* transcript levels. In non-PBD cells, this accounted for 28.3 % of total *PEX1* transcripts. Notably, all patient cell lines had some canonical *PEX1* transcript. This likely resulted from “leaky” splice site mutations that allowed some normal transcript to be made. In patient 1, this accounted for 11.5 %, patient 2, 5.2 % and patient 3, 1.5 % of total *PEX1* transcripts. In Patient 3, a novel transcript involving exon 16 skipping was identified and accounted for 68.9 % of total PEX1 transcripts (Supplemental Table 1).

#### 3.4.2. Evaluation of PEX1 transcript isoforms

**Fig. 3.**
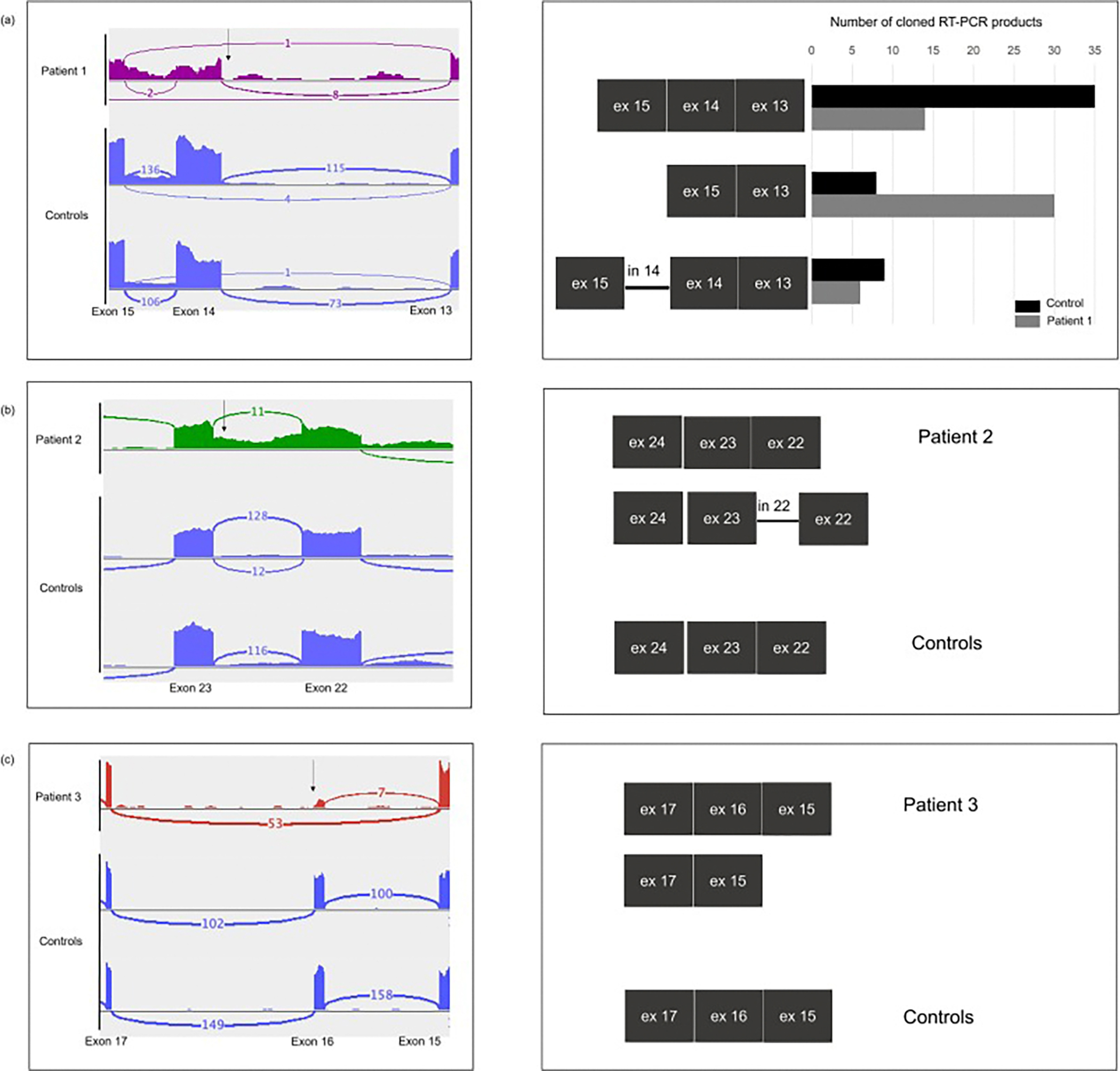
PEX1 transcript analysis showing aberrant PEX1 splicing in patient fibroblasts. (a) Patient 1, left: Sashimi plot of RNA-seq analysis showed exon 14 skipping and intron 14 retention in both Patient 1 and control cell lines. Arrow indicates position of c.2227–11 T > A variant. Patient 1, right: Schematic of transcripts detected (data shown in Supplementary Figs. 1 and 4). RT-PCR using an exon12/exon15 primer pair was performed, and 52 control and 50 patient clones were obtained. Cloned fragments were of 3 different sizes corresponding to exon 13–14–15, exon 14 skipping, and intron 14 retention. Number of clones containing each of the three fragments is shown. Sanger sequencing showed exon 14 skipping occurred in trans with the c.2097dupT variant. Retention of intron 14 was observed in transcripts containing c.2097dupT and those without this variant. (b) Patient 2, left: RNA-seq analysis identified intron 22 retention and a novel variant c.3637–19 A > G (arrow) within intron 22 in Patient 2; Sashimi plot shown. Patient 2, right: Schematic of transcripts detected (data shown in Supplementary Figs. 2 and 5): RT-PCR using exon22/exon24 primer pair was performed and showed 2 bands corresponding to exon 22–23–24 and a larger band corresponding to exon 22-intron 22-exon 23 –exon 24. Sanger sequencing of RT-PCR clones (exon22/exon24 primer pair) confirmed the presence of the c.3637–19 A > G variant along with complete retention of intron 22 and showed that c.3637–19 A > G was In trans with the second allele, c.2916del A. The two variants were heterozygous in the genomic DNA. (c) Patient 3, left: Sashimi plot of RNA-seq analysis identified exon 16 skipping in Patient 3. Arrow indicates position of c.2718G > A variant. Patient 3, right: Schematic of transcripts detected: Sanger sequencing of RT-PCR products using exon 15/exon 17 primer pair confirmed complete exon 16 skipping.

Fig. 3 shows the Sashimi plots from RNA-seq analysis in the left column and the corresponding schema of the transcripts detected by RT-PCR and sequencing in the right column (PCR products and sanger sequencing results are shown in supplementary figures). In Patient 1 (c.2097dupT/ c.2227–11 T > A), RNA-seq showed 1 read supporting exon 14 skipping as well as evidence of intron 14 retention both of which were also detected in controls (Fig. 3a, left). PCR amplification using an exon13/exon15 primer pair showed two fragments in Patient 1, one corresponding to controls (450 bp) and a second smaller fragment (260 bp) (Supplementary fig. 1a). Cloning of 102 products from an independent RT-PCR using exon12/exon15 primer pair showed 3 different fragments in both Patient 1 and control. Sanger sequencing of these clones showed that the largest fragment contained complete retention of intron 14 (863 bp), the smallest fragment (60 % of Patient 1 fragments, 15.3 % of control fragments) had deletion of exon 14 (457 bp), and the middle fragment had the normal exonic sequence (exons 13, 14, 15; 647 bp) (schematic Fig. 3a right; Supplemental fig. 1b). Sanger sequencing of cloned fragments revealed that the c.2097dupT variant was *in trans* with the transcript in which exon 14 was skipped (Supplementary Fig. 4). Altogether, the RT-PCR data suggest that the c.2227–11 T > A variant increases the proportion of transcripts with exon 14 skipping. This shifts the reading frame and generates a premature stop codon in exon 15 at position c.2420 (TAG), likely resulting in nonsense-mediated decay (NMD), as reflected by the low level of *PEX1* transcripts seen in RNA-seq.

In Patient 2, RNA-seq (Fig. 3b left) showed a novel c.3637–19 A > G variant in intron 22 and 19 reads supporting intron 22 retention. To evaluate this, we performed RT-PCR using an exon22/exon24 primer pair and identified 2 fragments, one the same size as control (846 bp) and a larger fragment (1136 bp) (Supplemental fig. 2). Sanger sequencing of the cloned fragments revealed complete retention of intron 22 in the larger fragment along with the c.3637–19 A > G variant in intron 22 (schematic Fig. 3b right). Additional RT-PCR using an exon18/23 primer pair and sequencing of the products revealed that the products containing the intronic variant did not contain the c.2916delA variant previously identified on WES, thus demonstrating that the two variants are *in trans*. Finally, the c.3637–19 A > G variant in intron 22 was heterozygous in genomic DNA (Supplementary Fig. 5). In silico analysis of the intronic variant using Human Splice Finder predicted activation of a cryptic splice site (+52,01 %), and SpliceAI predicted an acceptor gain (Δ score 0.75). There were also 11 RNA-seq reads that supported normal exon 22/exon 23 junctions (Fig. 3b left). This finding, together with the very low amounts of canonical PEX1 transcript (0.068764 FPKM), suggests that the c.3637–19 A > G variant causes leaky splicing, resulting in transcripts with intron 22 retention and production of some normal transcripts. A premature stop codon in intron 22 at position c.3636 + 31 (TAA) likely results in transcript degradation by NMD.

In Patient 3, RNA-seq (Fig. 3c left) identified the c.2718G > A variant found on WES and showed evidence of exon 16 skipping supported by 53 reads. This aberrantly spliced transcript was the predominantly expressed PEX1 transcript in Patient 3 (1.962446 FPKM), compared to very low amounts of the canonical PEX1 transcript (0.043829 FPKM). To corroborate this finding, RT-PCR using an exon15/exon17 primer pair showed both a faint band corresponding to a normal transcript (350 bp), and a strong band of lower molecular weight, corresponding to the absence of exon 16 (215 bp) (Supplemental fig. 3). Sanger sequencing of RT-PCR products confirmed complete exon16 skipping in the patient (schematic Fig. 3c right, and Supplementary Fig. 6).

### 3.5. PEX1, PEX6, and PEX5 protein amounts

**Fig. 4.**
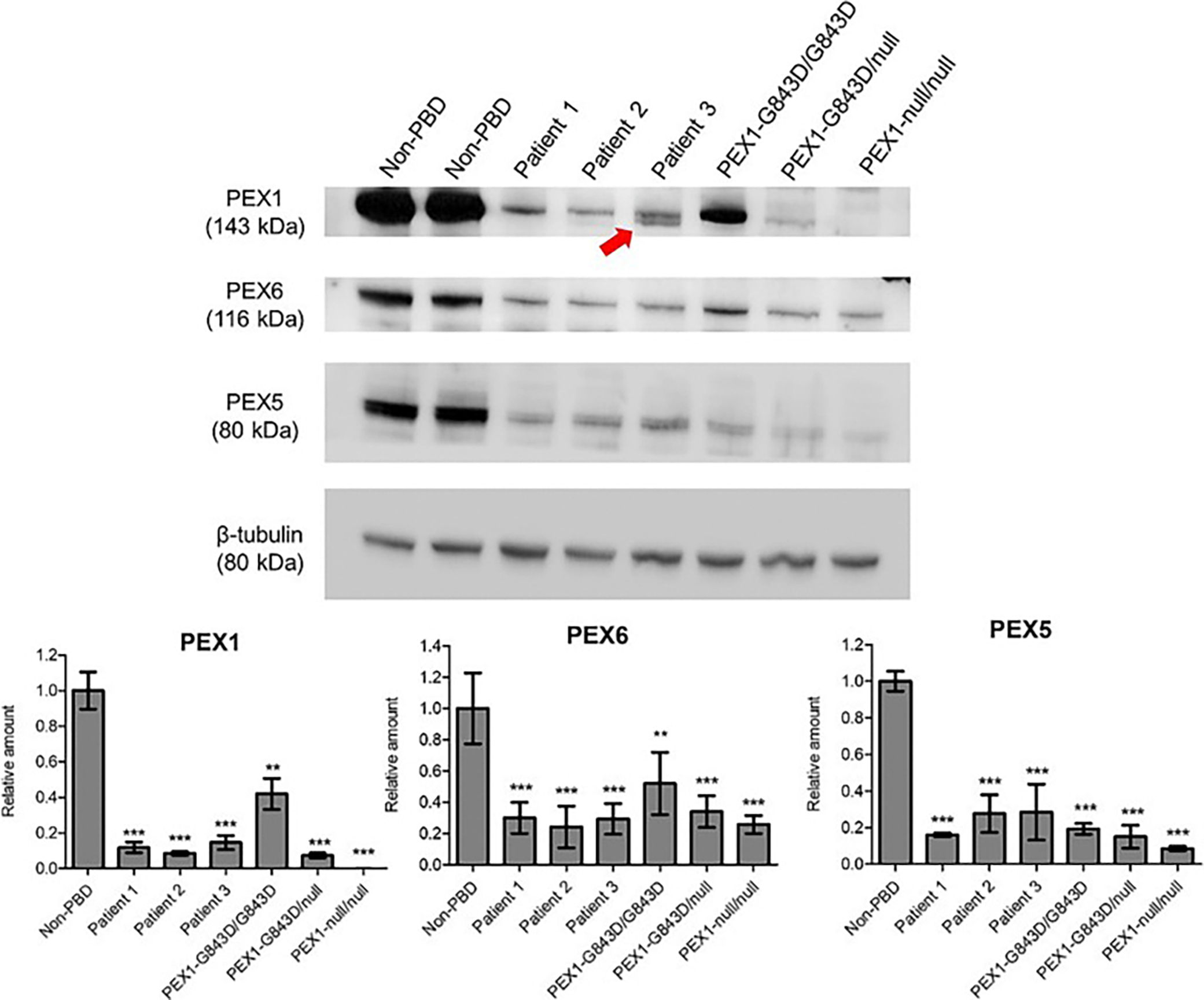
Amounts of PEX1, PEX6 and PEX5 protein in primary fibroblast cell lines from Patients 1, 2 and 3. Immunoblotting of cell lysates showed reduced PEX1 protein in all patient fibroblasts compared to non-PBD controls. In Patient 3, two PEX1 bands were seen, one corresponding to full length PEX1 (143 kDa, on average 65 % of total PEX1) and the other ~138 kDa (red arrow, on average 35 % of total PEX1), corresponding to the absence of the 45 amino acids in exon 16. PEX6 and PEX5 were also reduced in patient cell lines compared to non-PBD controls. Primary patient fibroblast lines with the common ZSD genotypes PEX1-p. Gly843Asp/Gly843Asp, Gly843Asp/null, and null/null were included for comparison. β-tubulin was used as a loading control. Histograms show band densitometry quantification normalized to loading control and relative to non-PBD average (mean ± SD). *N* = 3; 1-way ANOVA with Neuman-Keuls post-test; ** = *p* ≤ 0.01, *** = *p* ≤ 0.001 compared to non-PBD control.

It is known that PEX1 deficiency causes reduced amounts of its partner protein PEX6 and reduced amounts of PEX5 due to failure to remove PEX5 from the peroxisomal membrane by the PEX1/6 complex. Accumulated PEX5 on the peroxisomal membrane is subsequently degraded [10,24]. Thus, we evaluated levels of PEX1, PEX6 and PEX5 via immunoblotting of whole cell lysates from patient fibroblast cultures (Fig. 4). Two non-PBD controls and primary fibroblasts from patients with PEX1-p.Gly843Asp/ Gly 843Asp, (mild phenotype), PEX1-p. Gly843Asp / Gln128* (intermediate phenotype) and PEX1-p. Ile700Tyrfs*42/ Ile700Tyrfs*42 (severe phenotype) were included for comparison. PEX1 amounts were decreased to 8–15 % of normal (non-PBD) levels, on average, in all three patient cell lines. These values fall between those of the p. Gly843Asp/ Gly843Asp and p. Gly843Asp / Gln128* cell lines, which showed 42 % and 7 % of normal PEX1 levels, respectively. As expected, PEX1 protein was not observed in the PEX1-p. Ile700Tyrfs*42/ Ile700Tyrfs*42 (null) cell line. Two PEX1 bands were observed in Patient 3, one corresponding to normal PEX1 protein and making up 65 % of the total PEX1 detected, and the other of lower molecular weight corresponding to the lack of 45 amino acids contained in exon 16 (~5 kDa), accounting for 35 % of PEX1 protein detected in this line. PEX6 and PEX5 levels were decreased to 16–28 % of the non-PBD average, close to or higher than PEX5 levels in the PEX1-p. Gly843Asp/ Gly843Asp cell line (19 % of normal).

## 4. Discussion

Next generation sequencing, in the form of gene panels and WES, has become the definitive diagnostic test for ZSD. In cases of suspected ZSD with inconclusive WES, traditional biochemical testing and functional assays on patient fibroblasts may help identify peroxisome dysfunction and support the need for further investigation of peroxisomal disorder genes. This was the case for our patients where identification of *PEX1* variants of uncertain significance (Patients 1 and 3) and a single *PEX1* pathogenic variant (Patient 2), together with abnormalities in peroxisome metabolites, led to the pursuit of peroxisome functional studies and *PEX1* RNA analysis.

The lack of coverage of non-coding regions remains a major limitation of gene panels and WES. As such, RNA-seq has been proposed as a complementary tool to WES in the diagnosis of monogenic disorders [25,26]. The recent report of a deep intronic PEX1 variant found by RNA-seq in a patient with mild ZSD highlights the utility of this modality in the diagnosis of ZSD [11]. However, RNA-seq has its limitations. These include low read depths resulting from NMD and biological samples in which targeted gene expression is low causing missed aberrant splicing events. Furthermore, due to short reads, there is difficulty to phase single nucleotide variants and their corresponding aberrant splicing events [27]. RT-PCR followed by Sanger sequencing offers a higher sensitivity and is thus useful as a complimentary test to validate RNA-seq. This was the case for Patient 1, where RNA-seq data only showed 1 supporting read for exon 14 skipping and failed to identify the c.2227–11 T > A variant due to low read depth, likely a result of NMD. Although increasing sequence depth may help reveal aberrant splicing events, our study already had a total sequence depth of 240–380 million reads per patient sample. When we proceeded with RT-PCR and Sanger sequencing of that region, the shorter products lacking exon 14 were likely preferentially amplified thus revealing the mis-splicing event.

Our series of patients all had a non-severe ZSD phenotype. This is possibly due to the presence of very low amounts of canonical *PEX1* transcripts on RNA-seq and the presence of full length PEX1 protein in their fibroblasts. This is highlighted in the case of Patient 2 who harbored a p. Gly973Alafs*16 truncating variant and the intronic variant c.3637–19 A > G that causes retention of intron 22. Patients who are compound heterozygotes for the former and a different truncating variant are reported to have severe phenotypes with cell lines showing no detectable PEX1 protein or functional peroxisomes [28,29]. However, our patient had an intermediate phenotype with survival until 3 years of age and a milder cellular phenotype with residual import functions and residual PEX1 protein. Her intermediate phenotype is likely due to production of residual PEX1 via leaky splicing from the c.3637–19 A > G intronic variant.

The presence of both full-length PEX1 and PEX1 lacking exon 16 likely contributes to the very mild clinical and cellular phenotype of Patient 3. Given that complete exon 16 skipping maintains the reading frame, there may be residual function in the resultant shorter PEX1 protein. Alternatively, the PEX1 protein with exon 16 skipping may compete with normal PEX1 to form the PEX1-PEX6 complex. The incorporation of this abnormal PEX1 protein may interfere with the function of the PEX1-PEX6 complex. Indeed, exon 16 contains the Walker A motif (GPPGTGKT) in the D2 ATPase domain which is required for ATP binding as part of the heterohexameric AAA-ATPase complex formed by PEX1 and PEX6 [30].

When applying the American College of Medical Genetics variant interpretation guidelines and recent recommendations for interpretation of splicing data, we were able to classify the novel variant in Patients 1 and 2 as likely pathogenic, but unable to reclassify the VUS in Patient 3 [31,32]. However, this VUS is categorized as “Warm” [33]. As such, it may be reclassified as likely pathogenic/pathogenic if further evidence of pathogenicity arises. See Supplemental Table 2 for our detailed interpretation of these variants.

## 5. Conclusion

In 3 patients with suspected *PEX1*-related ZSD, we confirmed peroxisomal dysfunction using functional assays in fibroblasts and demonstrated partial splicing defects in *PEX1* on RNA studies. These investigations ultimately confirmed a diagnosis of ZSD in these patients, highlighting the utility of multiple diagnostic modalities in the workup of patients with inconclusive WES.

## Supplementary Material

Supplemental Data

Appendix A. Supplementary data

Supplementary data to this article can be found online at https://doi.org/10.1016/j.ymgme.2025.109080.

## Figures and Tables

**Table 1.** Patient clinical features.

Empty Cell	Pt1	Pt2	Pt3

**Sex (current age in years)**	M (36)	F (deceased:3)	F (10)
**Age at confirmed diagnosis (years)**	34	2.5	9
***PEX1* variants**	c.2097dupT / 2227–11 T > A	c.2916delA / 3637–19 A > G	c.2718G > A / 2718G > A
**Predicted PEX1 protein**	p.I700Yfs*42 / -	p.G973Afs*16 / -	p.K906(=) / K906(=)
**Neurological abnormalities**	Progressive generalized dystonia, spasticity, ataxia	Hypotonia, hyperreflexia	Learning disorder, inattention, hyperactivity
**Seizures**	−	Infantile myoclonic epilepsy	−
**Global developmental delay**	+	+	−
**Hearing impairment**	+	+	+
**Vision impairment**	−	+	−
**Cataracts**	−	−	−
**Liver abnormality**	−	−	−
**Adrenal insufficiency**	−	ND	ND
**Nephrolithiasis**	−	−	−
**Amelogenesis Imperfecta**	−	?poor oral hygiene, small conical and abnormally spaced teeth	−
**Brain MRI findings (age in years)**	Pontocerebellar atrophy and pontine cruciform signal abnormality (26)	Ventriculomegaly (1)	Normal (4.5)

Pt, patient; M, male; F, female; (+) feature present; (−) feature not present; ND, no data.

**Table 2.** Peroxisome metabolite levels.

Peroxisome metabolites in blood	Pt1 (31 yo)	Pt1 (32 yo)	Pt2 (1 yo)	Pt2 (1.5 yo)	Reference range

C26:0 (ug/ml)	0.39 ↑	ND	0.57 ↑↑	0.74 ↑↑	0.23 ± 0.09
C24:0/C22:0	0.973 ↑	ND	1.17 ↑↑	1.22 ↑↑	0.84 ± 0.10
C26:0/C22:0	0.019 ↑↑	ND	0.046 ↑↑	0.049	0.01 ± 0.004
Phytanic acid (ug/ml)	0.8	ND	1.45	2.91	<3.0
Pristanic acid (ug/ml)	0.07	ND	0.107	0.59 ↑	<0.3
Pipecolic acid (ug/ml)	ND	0.142	ND	ND	0.01–0.52
Plasmalogens C18:0 DMA / C18:0¶	0.182 ↓	0.179 ↓	ND	0.157↓	0.199–0.284
Plasmalogens C16:0 DMA /C16:0¶	0.105	0.103	ND	0.079	0.079–0.128
Peroxisome metabolites in fibroblasts	**Pt3 (9 yo)**				
C26:0 LysoPC (fold over control)	2.5				
Total PE Plasmalogens (fold over control)	0.83				

Pt, patient; yo, years-old; ND, no data; DMA, dimethylacetals; ¶,Measured in erythrocytes; PC, phosphatidylcholine; PE, phosphatidylethanolamine; C26:0, C24:0/C22:0, C26:0/C22?0, ↑ one STD above normal, ↑↑ two STD above normal.

## Data Availability

Data will be made available on request.

## Abbreviations:

FPKM: fragments per kilobase per million mapped reads
LC-MS/MS: liquid chromatography-tandem mass spectrometry
lyso-PC: lysophosphatidylcholine
NGS: next generation sequencing
NMD: nonsense-mediated decay
PBD: peroxisome biogenesis disorder
RNA-seq: RNA sequencing
RT-PCR: reverse transcription-polymerase chain reaction
SNHL: sensorineural hearing loss
VLCFAs: very long chain fatty acids
VUS: variant of uncertain significance
WES: whole exome sequencing
ZSD: Zellweger spectrum disorder
