# Supplementary material for: Using multiple modalities to confirm diagnosis in patients with suspected peroxisome biogenesis disorders
Source: Mol Genet Metab. Author manuscript; Available in PMC 2026 Mar 11. (PMC12301650; doi:10.1016/j.ymgme.2025.109080)
Supplement: Supplemental Data [file NIHMS2095912-supplement-Supplemental_Data.pptx]

## Slide 1
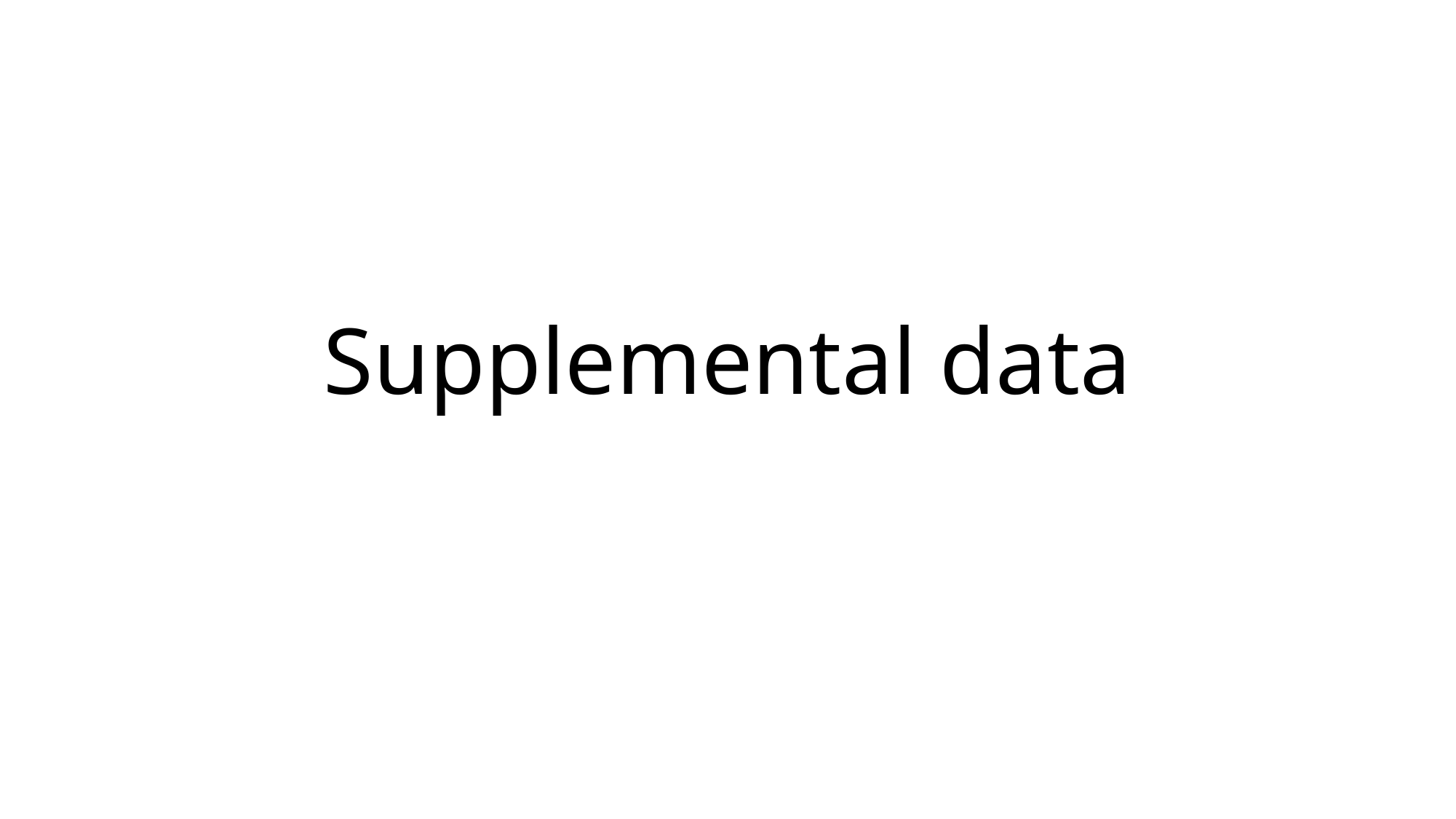

# Supplemental data

## Slide 2
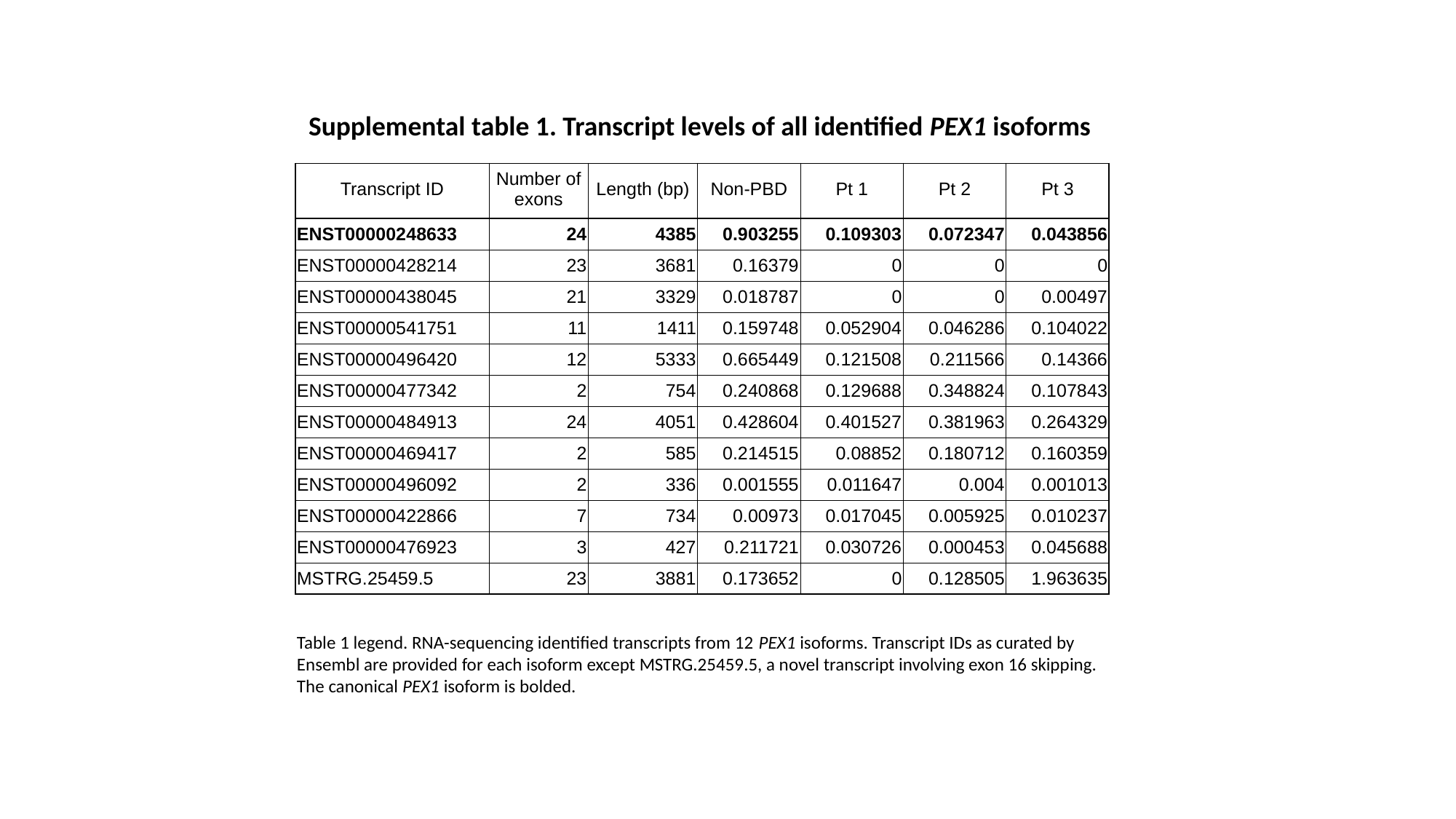

Supplemental table 1. Transcript levels of all identified PEX1 isoforms
| Transcript ID | Number of exons | Length (bp) | Non-PBD | Pt 1 | Pt 2 | Pt 3 |
| --- | --- | --- | --- | --- | --- | --- |
| ENST00000248633 | 24 | 4385 | 0.903255 | 0.109303 | 0.072347 | 0.043856 |
| ENST00000428214 | 23 | 3681 | 0.16379 | 0 | 0 | 0 |
| ENST00000438045 | 21 | 3329 | 0.018787 | 0 | 0 | 0.00497 |
| ENST00000541751 | 11 | 1411 | 0.159748 | 0.052904 | 0.046286 | 0.104022 |
| ENST00000496420 | 12 | 5333 | 0.665449 | 0.121508 | 0.211566 | 0.14366 |
| ENST00000477342 | 2 | 754 | 0.240868 | 0.129688 | 0.348824 | 0.107843 |
| ENST00000484913 | 24 | 4051 | 0.428604 | 0.401527 | 0.381963 | 0.264329 |
| ENST00000469417 | 2 | 585 | 0.214515 | 0.08852 | 0.180712 | 0.160359 |
| ENST00000496092 | 2 | 336 | 0.001555 | 0.011647 | 0.004 | 0.001013 |
| ENST00000422866 | 7 | 734 | 0.00973 | 0.017045 | 0.005925 | 0.010237 |
| ENST00000476923 | 3 | 427 | 0.211721 | 0.030726 | 0.000453 | 0.045688 |
| MSTRG.25459.5 | 23 | 3881 | 0.173652 | 0 | 0.128505 | 1.963635 |
Table 1 legend. RNA-sequencing identified transcripts from 12 PEX1 isoforms. Transcript IDs as curated by Ensembl are provided for each isoform except MSTRG.25459.5, a novel transcript involving exon 16 skipping. The canonical PEX1 isoform is bolded.

## Slide 3
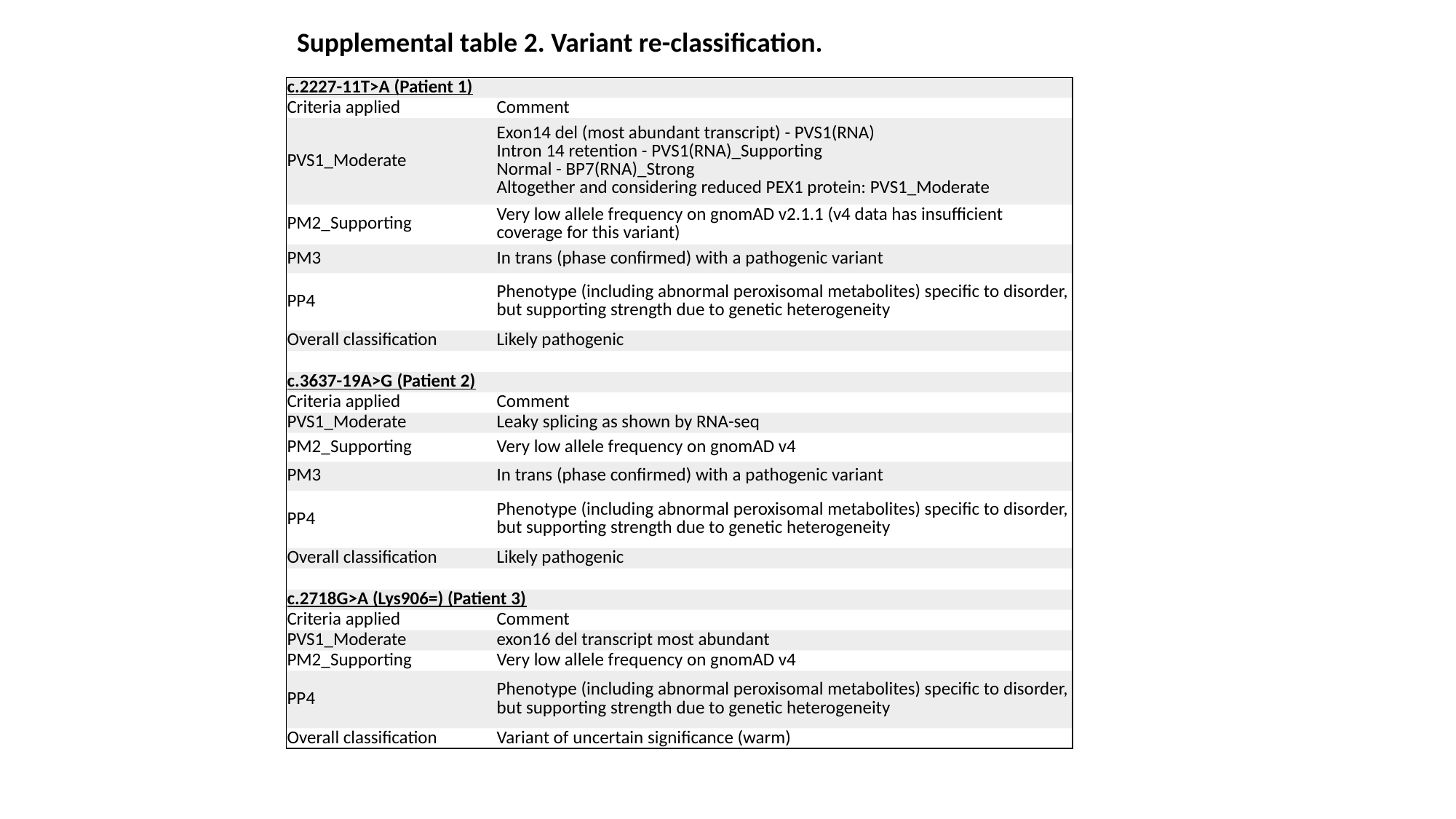

Supplemental table 2. Variant re-classification.
| c.2227-11T>A (Patient 1) | |
| --- | --- |
| Criteria applied | Comment |
| PVS1\_Moderate | Exon14 del (most abundant transcript) - PVS1(RNA) Intron 14 retention - PVS1(RNA)\_Supporting Normal - BP7(RNA)\_Strong Altogether and considering reduced PEX1 protein: PVS1\_Moderate |
| PM2\_Supporting | Very low allele frequency on gnomAD v2.1.1 (v4 data has insufficient coverage for this variant) |
| PM3 | In trans (phase confirmed) with a pathogenic variant |
| PP4 | Phenotype (including abnormal peroxisomal metabolites) specific to disorder, but supporting strength due to genetic heterogeneity |
| Overall classification | Likely pathogenic |
| | |
| c.3637-19A>G (Patient 2) | |
| Criteria applied | Comment |
| PVS1\_Moderate | Leaky splicing as shown by RNA-seq |
| PM2\_Supporting | Very low allele frequency on gnomAD v4 |
| PM3 | In trans (phase confirmed) with a pathogenic variant |
| PP4 | Phenotype (including abnormal peroxisomal metabolites) specific to disorder, but supporting strength due to genetic heterogeneity |
| Overall classification | Likely pathogenic |
| | |
| c.2718G>A (Lys906=) (Patient 3) | |
| Criteria applied | Comment |
| PVS1\_Moderate | exon16 del transcript most abundant |
| PM2\_Supporting | Very low allele frequency on gnomAD v4 |
| PP4 | Phenotype (including abnormal peroxisomal metabolites) specific to disorder, but supporting strength due to genetic heterogeneity |
| Overall classification | Variant of uncertain significance (warm) |

## Slide 4
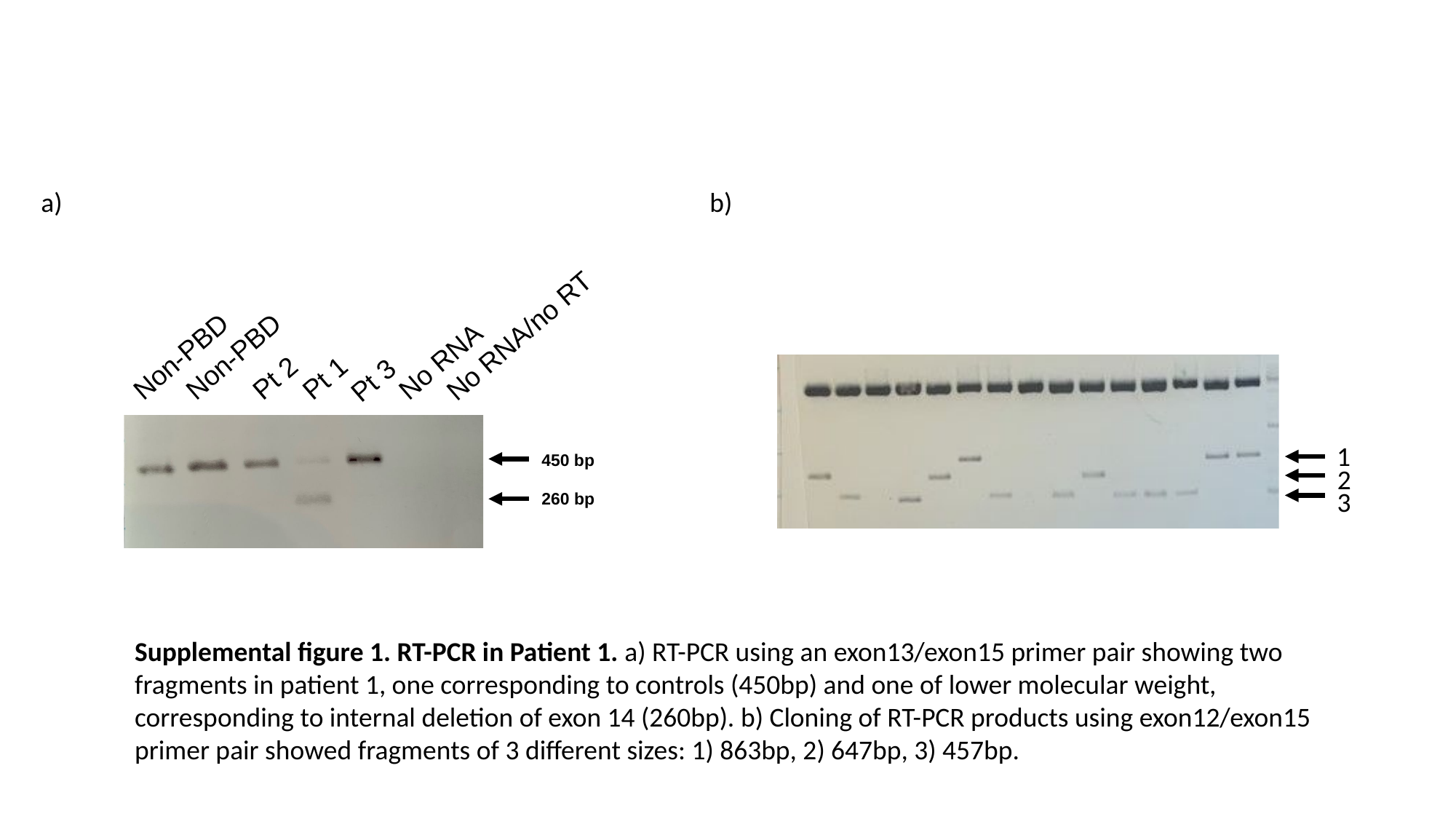

a)
b)
No RNA
No RNA/no RT
Non-PBD
Non-PBD
Pt 2
Pt 1
Pt 3
1
450 bp
2
3
260 bp
Supplemental figure 1. RT-PCR in Patient 1. a) RT-PCR using an exon13/exon15 primer pair showing two fragments in patient 1, one corresponding to controls (450bp) and one of lower molecular weight, corresponding to internal deletion of exon 14 (260bp). b) Cloning of RT-PCR products using exon12/exon15 primer pair showed fragments of 3 different sizes: 1) 863bp, 2) 647bp, 3) 457bp.

## Slide 5
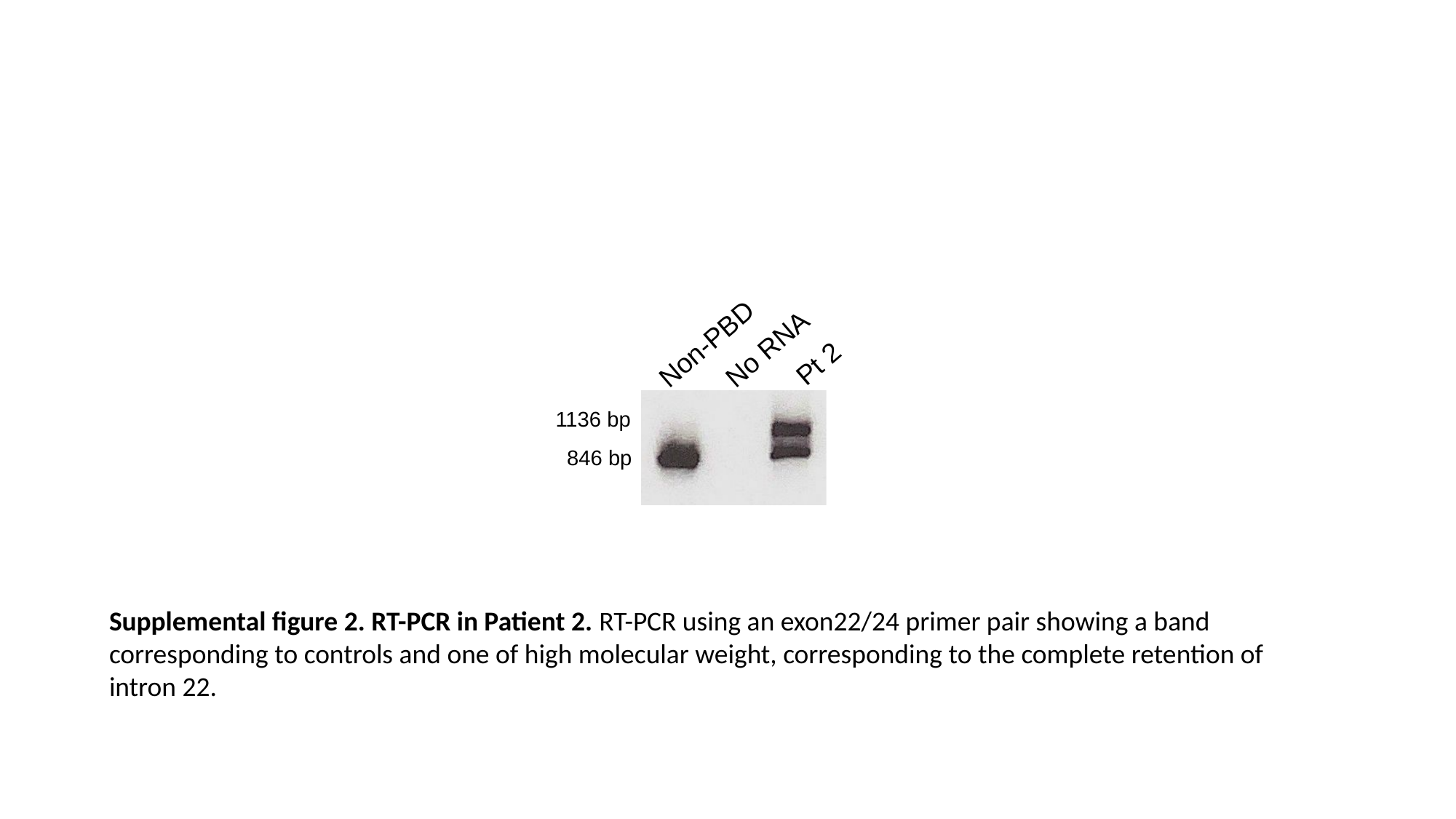

No RNA
Non-PBD
Pt 2
1136 bp
846 bp
Supplemental figure 2. RT-PCR in Patient 2. RT-PCR using an exon22/24 primer pair showing a band corresponding to controls and one of high molecular weight, corresponding to the complete retention of intron 22.

## Slide 6
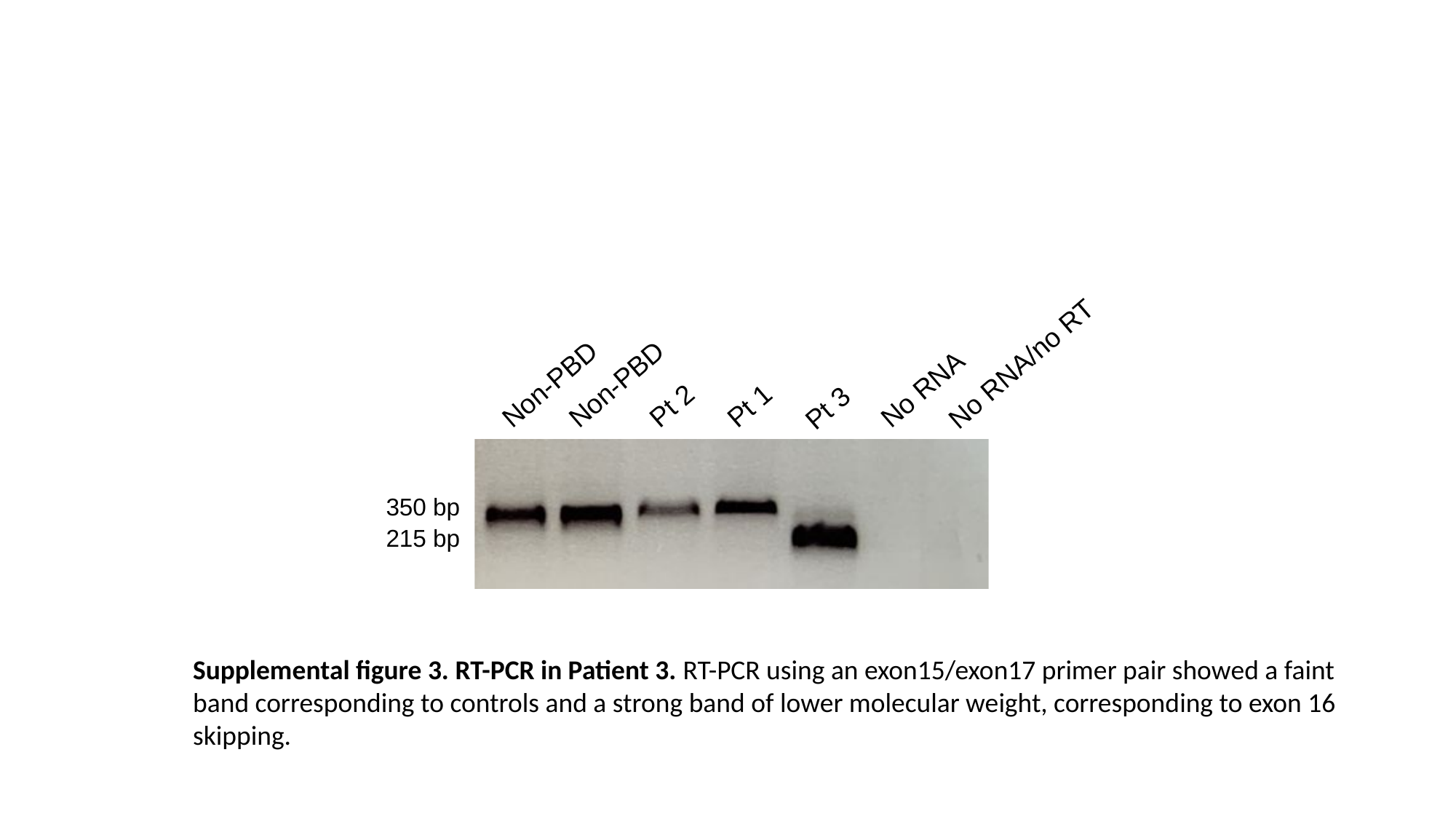

No RNA
No RNA/no RT
Non-PBD
Non-PBD
Pt 2
Pt 1
Pt 3
350 bp
215 bp
Supplemental figure 3. RT-PCR in Patient 3. RT-PCR using an exon15/exon17 primer pair showed a faint band corresponding to controls and a strong band of lower molecular weight, corresponding to exon 16 skipping.

## Slide 7
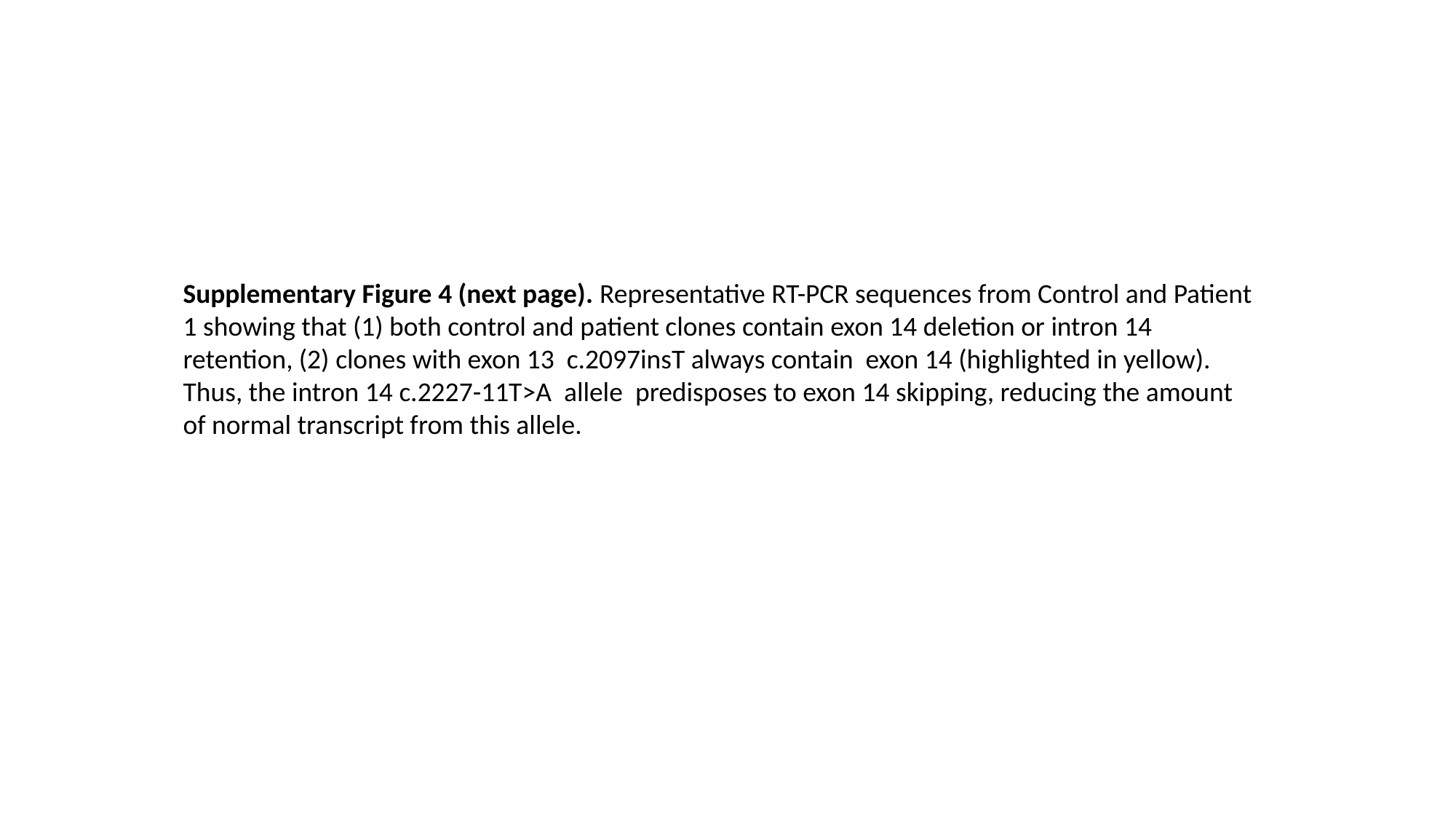

Supplementary Figure 4 (next page). Representative RT-PCR sequences from Control and Patient 1 showing that (1) both control and patient clones contain exon 14 deletion or intron 14 retention, (2) clones with exon 13 c.2097insT always contain exon 14 (highlighted in yellow). Thus, the intron 14 c.2227-11T>A allele predisposes to exon 14 skipping, reducing the amount of normal transcript from this allele.

## Slide 8
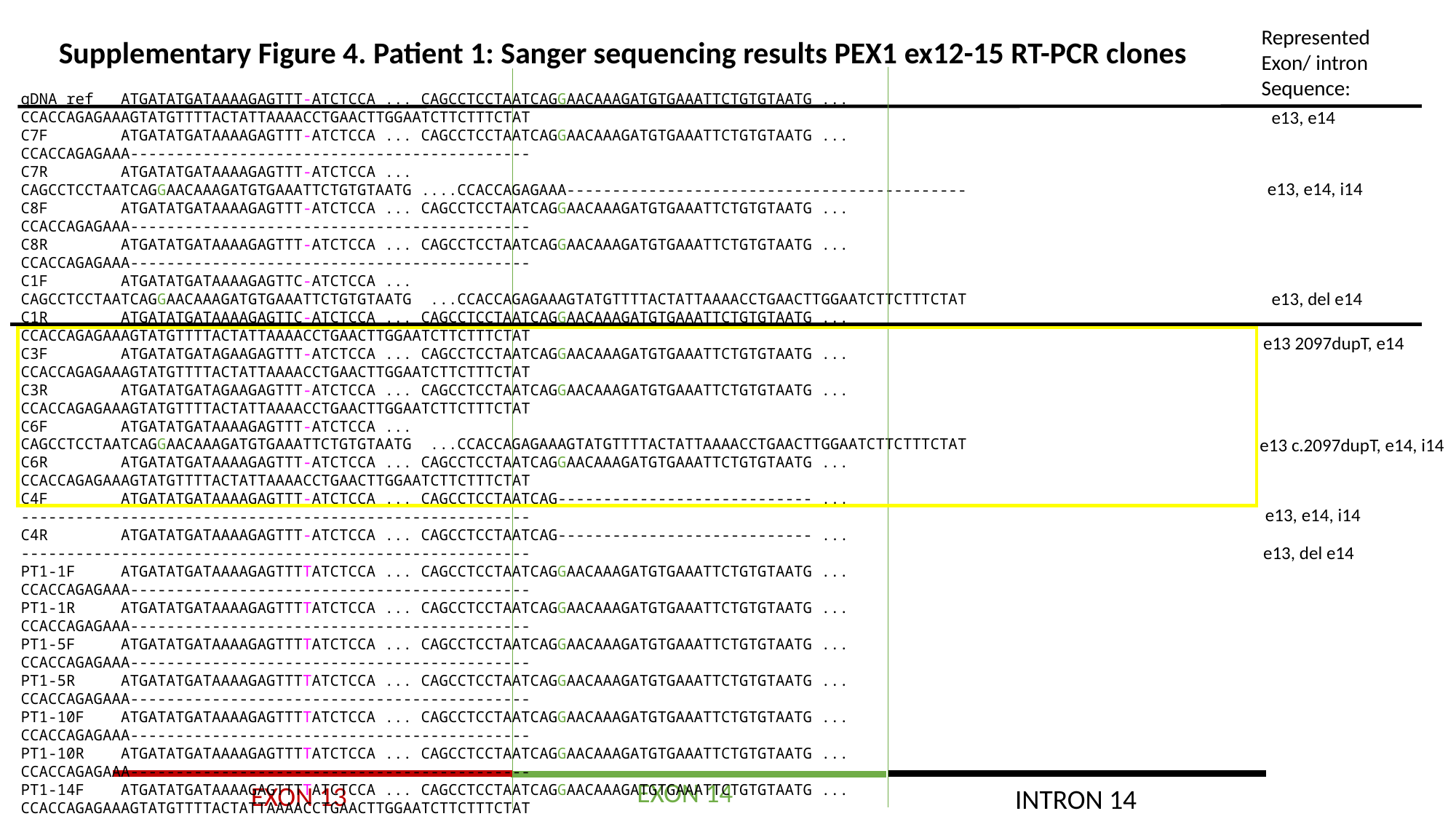

Represented
Exon/ intron Sequence:
# Supplementary Figure 4. Patient 1: Sanger sequencing results PEX1 ex12-15 RT-PCR clones
gDNA ref ATGATATGATAAAAGAGTTT-ATCTCCA ... CAGCCTCCTAATCAGGAACAAAGATGTGAAATTCTGTGTAATG ... CCACCAGAGAAAGTATGTTTTACTATTAAAACCTGAACTTGGAATCTTCTTTCTAT
C7F ATGATATGATAAAAGAGTTT-ATCTCCA ... CAGCCTCCTAATCAGGAACAAAGATGTGAAATTCTGTGTAATG ... CCACCAGAGAAA--------------------------------------------
C7R ATGATATGATAAAAGAGTTT-ATCTCCA ... CAGCCTCCTAATCAGGAACAAAGATGTGAAATTCTGTGTAATG ....CCACCAGAGAAA--------------------------------------------
C8F ATGATATGATAAAAGAGTTT-ATCTCCA ... CAGCCTCCTAATCAGGAACAAAGATGTGAAATTCTGTGTAATG ... CCACCAGAGAAA--------------------------------------------
C8R ATGATATGATAAAAGAGTTT-ATCTCCA ... CAGCCTCCTAATCAGGAACAAAGATGTGAAATTCTGTGTAATG ... CCACCAGAGAAA--------------------------------------------
C1F ATGATATGATAAAAGAGTTC-ATCTCCA ... CAGCCTCCTAATCAGGAACAAAGATGTGAAATTCTGTGTAATG ...CCACCAGAGAAAGTATGTTTTACTATTAAAACCTGAACTTGGAATCTTCTTTCTAT
C1R ATGATATGATAAAAGAGTTC-ATCTCCA ... CAGCCTCCTAATCAGGAACAAAGATGTGAAATTCTGTGTAATG ... CCACCAGAGAAAGTATGTTTTACTATTAAAACCTGAACTTGGAATCTTCTTTCTAT
C3F ATGATATGATAGAAGAGTTT-ATCTCCA ... CAGCCTCCTAATCAGGAACAAAGATGTGAAATTCTGTGTAATG ... CCACCAGAGAAAGTATGTTTTACTATTAAAACCTGAACTTGGAATCTTCTTTCTAT
C3R ATGATATGATAGAAGAGTTT-ATCTCCA ... CAGCCTCCTAATCAGGAACAAAGATGTGAAATTCTGTGTAATG ... CCACCAGAGAAAGTATGTTTTACTATTAAAACCTGAACTTGGAATCTTCTTTCTAT
C6F ATGATATGATAAAAGAGTTT-ATCTCCA ... CAGCCTCCTAATCAGGAACAAAGATGTGAAATTCTGTGTAATG ...CCACCAGAGAAAGTATGTTTTACTATTAAAACCTGAACTTGGAATCTTCTTTCTAT
C6R ATGATATGATAAAAGAGTTT-ATCTCCA ... CAGCCTCCTAATCAGGAACAAAGATGTGAAATTCTGTGTAATG ... CCACCAGAGAAAGTATGTTTTACTATTAAAACCTGAACTTGGAATCTTCTTTCTAT
C4F ATGATATGATAAAAGAGTTT-ATCTCCA ... CAGCCTCCTAATCAG---------------------------- ... --------------------------------------------------------
C4R ATGATATGATAAAAGAGTTT-ATCTCCA ... CAGCCTCCTAATCAG---------------------------- ... --------------------------------------------------------
PT1-1F ATGATATGATAAAAGAGTTTTATCTCCA ... CAGCCTCCTAATCAGGAACAAAGATGTGAAATTCTGTGTAATG ... CCACCAGAGAAA--------------------------------------------
PT1-1R ATGATATGATAAAAGAGTTTTATCTCCA ... CAGCCTCCTAATCAGGAACAAAGATGTGAAATTCTGTGTAATG ... CCACCAGAGAAA--------------------------------------------
PT1-5F ATGATATGATAAAAGAGTTTTATCTCCA ... CAGCCTCCTAATCAGGAACAAAGATGTGAAATTCTGTGTAATG ... CCACCAGAGAAA--------------------------------------------
PT1-5R ATGATATGATAAAAGAGTTTTATCTCCA ... CAGCCTCCTAATCAGGAACAAAGATGTGAAATTCTGTGTAATG ... CCACCAGAGAAA--------------------------------------------
PT1-10F ATGATATGATAAAAGAGTTTTATCTCCA ... CAGCCTCCTAATCAGGAACAAAGATGTGAAATTCTGTGTAATG ... CCACCAGAGAAA--------------------------------------------
PT1-10R ATGATATGATAAAAGAGTTTTATCTCCA ... CAGCCTCCTAATCAGGAACAAAGATGTGAAATTCTGTGTAATG ... CCACCAGAGAAA--------------------------------------------
PT1-14F ATGATATGATAAAAGAGTTTTATCTCCA ... CAGCCTCCTAATCAGGAACAAAGATGTGAAATTCTGTGTAATG ... CCACCAGAGAAAGTATGTTTTACTATTAAAACCTGAACTTGGAATCTTCTTTCTAT
PT1-14R ATGATATGATAAAAGAGTTTTATCTCCA ... CAGCCTCCTAATCAGGAACAAAGATGTGAAATTCTGTGTAATG ... CCACCAGAGAAAGTATGTTTTACTATTAAAACCTGAACTTGGAATCTTCTTTCTAT
PT1-15F ATGATATGATAAAAGAGTTTTATCTCCA ... CAGCCTCCTAATCAGGAACAAAGATGTGAAATTCTGTGTAATG ... CCACCAGAGAAAGTATGTTTTACTATTAAAACCTGAACTTGGAATCTTCTTTCTAT
PT1-15R ATGATATGATAAAAGAGTTTTATCTCCA ... CAGCCTCCTAATCAGGAACAAAGATGTGAAATTCTGTGTAATG ... CCACCAGAGAAAGTATGTTTTACTATTAAAACCTGAACTTGGAATCTTCTTTCTAT
PT1-6F ATGATATGATAAAAGAGTTT-ATCTCCA ... CAGCCTCCTAATCAGGAACAAAGATGTGAAATTCTGTGTAATG ... CCACCAGAGAAAGTATGTTTTACTATTAAAACCTGAACTTGGAATCTTCTTTCTAT
PT1-6R ATGATATGATAAAAGAGTTT-ATCTCCA ... CAGCCTCCTAATCAGGAACAAAGATGTGAAATTCTGTGTAATG ... CCACCAGAGAAAGTATGTTTTACTATTAAAACCTGAACTTGGAATCTTCTTTCTAT
PT1-2F ATGATATGATAAAAGAGTTT-ATCTCCA ... CAGCCTCCTAATCAG---------------------------- ... --------------------------------------------------------
PT1-2R ATGATATGATAAAAGAGTTT-ATCTCCA ... CAGCCTCCTAATCAG---------------------------- ... --------------------------------------------------------
PT1-7F ATGATATGATAAAAGAGTTT-ATCTCCA ... CAGCCTCCTAATCAG---------------------------- ... --------------------------------------------------------
PT1-7R ATGATATGATAAAAGAGTTT-ATCTCCA ... CAGCCTCCTAATCAG---------------------------- ... --------------------------------------------------------
PT1-9F ATGATATGATAAAAGAGTTT-ATCTCCA ... CAGCCTCCTAATCAG---------------------------- ... --------------------------------------------------------
PT1-9R ATGATATGATAAAAGAGTTT-ATCTCCA ... CAGCCTCCTAATCAG---------------------------- ... --------------------------------------------------------
PT1-11F ATGATATGATAAAAGAGTTT-ATCTCCA ... CAGCCTCCTAATCAG---------------------------- ... --------------------------------------------------------
PT1-11R ATGATATGATAAAAGAGTTT-ATCTCCA ... CAGCCTCCTAATCAG---------------------------- ... --------------------------------------------------------
PT1-12F ATGATATGATAAAAGAGTTT-ATCTCCA ... CAGCCTCCTAATCAG---------------------------- ... --------------------------------------------------------
PT1-12R ATGATATGATAAAAGAGTTT-ATCTCCA ... CAGCCTCCTAATCAG---------------------------- ... --------------------------------------------------------
PT1-13F ATGATATGATAGAAGAGTTT-ATCTCCA ... CAGCCTCCTAATCAG---------------------------- ... --------------------------------------------------------
PT1-13R ATGATATGATAGAAGAGTTT-ATCTCCA ... CAGCCTCCTAATCAG---------------------------- ... --------------------------------------------------------
e13, e14
e13, e14, i14
e13, del e14
e13 2097dupT, e14
e13 c.2097dupT, e14, i14
e13, e14, i14
e13, del e14
EXON 14
EXON 13
INTRON 14

## Slide 9
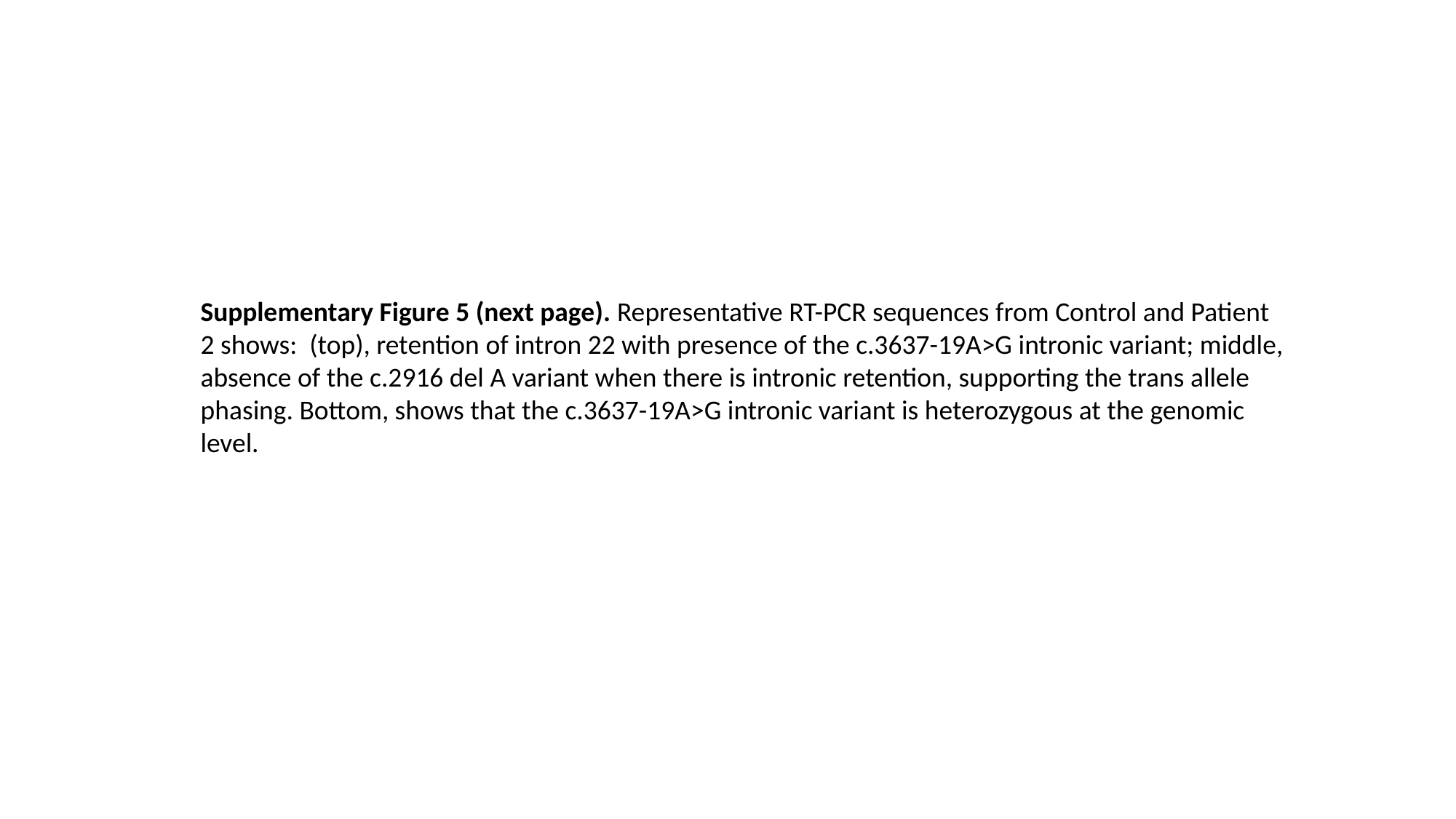

Supplementary Figure 5 (next page). Representative RT-PCR sequences from Control and Patient 2 shows: (top), retention of intron 22 with presence of the c.3637-19A>G intronic variant; middle, absence of the c.2916 del A variant when there is intronic retention, supporting the trans allele phasing. Bottom, shows that the c.3637-19A>G intronic variant is heterozygous at the genomic level.

## Slide 10
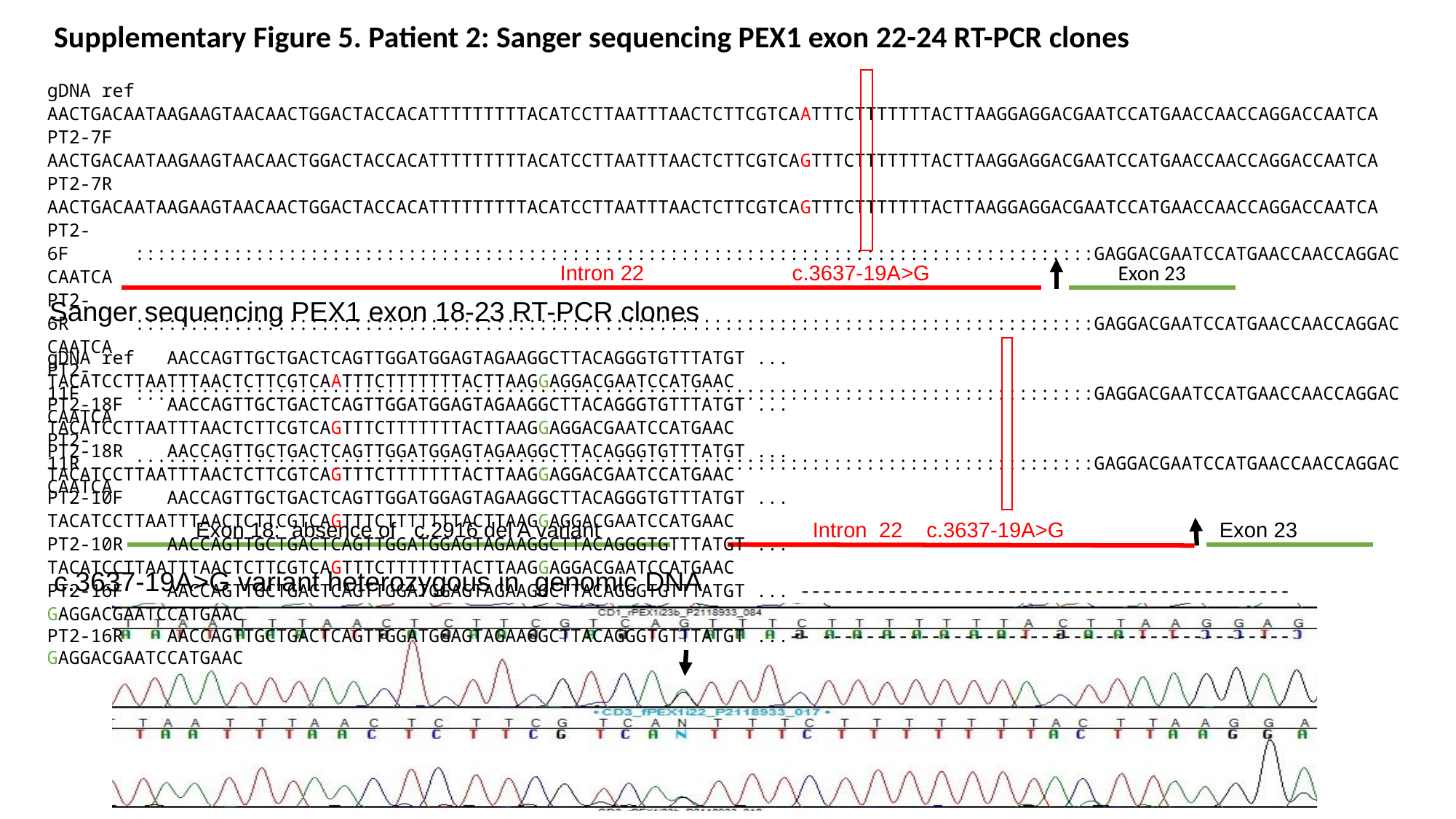

Supplementary Figure 5. Patient 2: Sanger sequencing PEX1 exon 22-24 RT-PCR clones
gDNA ref AACTGACAATAAGAAGTAACAACTGGACTACCACATTTTTTTTTACATCCTTAATTTAACTCTTCGTCAATTTCTTTTTTTACTTAAGGAGGACGAATCCATGAACCAACCAGGACCAATCA
PT2-7F AACTGACAATAAGAAGTAACAACTGGACTACCACATTTTTTTTTACATCCTTAATTTAACTCTTCGTCAGTTTCTTTTTTTACTTAAGGAGGACGAATCCATGAACCAACCAGGACCAATCA
PT2-7R AACTGACAATAAGAAGTAACAACTGGACTACCACATTTTTTTTTACATCCTTAATTTAACTCTTCGTCAGTTTCTTTTTTTACTTAAGGAGGACGAATCCATGAACCAACCAGGACCAATCA
PT2-6F ::::::::::::::::::::::::::::::::::::::::::::::::::::::::::::::::::::::::::::::::::::::::GAGGACGAATCCATGAACCAACCAGGACCAATCA
PT2-6R ::::::::::::::::::::::::::::::::::::::::::::::::::::::::::::::::::::::::::::::::::::::::GAGGACGAATCCATGAACCAACCAGGACCAATCA
PT2-11F ::::::::::::::::::::::::::::::::::::::::::::::::::::::::::::::::::::::::::::::::::::::::GAGGACGAATCCATGAACCAACCAGGACCAATCA
PT2-11R ::::::::::::::::::::::::::::::::::::::::::::::::::::::::::::::::::::::::::::::::::::::::GAGGACGAATCCATGAACCAACCAGGACCAATCA
Intron 22
c.3637-19A>G
Exon 23
Sanger sequencing PEX1 exon 18-23 RT-PCR clones
gDNA ref AACCAGTTGCTGACTCAGTTGGATGGAGTAGAAGGCTTACAGGGTGTTTATGT ... TACATCCTTAATTTAACTCTTCGTCAATTTCTTTTTTTACTTAAGGAGGACGAATCCATGAAC
PT2-18F AACCAGTTGCTGACTCAGTTGGATGGAGTAGAAGGCTTACAGGGTGTTTATGT ... TACATCCTTAATTTAACTCTTCGTCAGTTTCTTTTTTTACTTAAGGAGGACGAATCCATGAAC
PT2-18R AACCAGTTGCTGACTCAGTTGGATGGAGTAGAAGGCTTACAGGGTGTTTATGT ... TACATCCTTAATTTAACTCTTCGTCAGTTTCTTTTTTTACTTAAGGAGGACGAATCCATGAAC
PT2-10F AACCAGTTGCTGACTCAGTTGGATGGAGTAGAAGGCTTACAGGGTGTTTATGT ... TACATCCTTAATTTAACTCTTCGTCAGTTTCTTTTTTTACTTAAGGAGGACGAATCCATGAAC
PT2-10R AACCAGTTGCTGACTCAGTTGGATGGAGTAGAAGGCTTACAGGGTGTTTATGT ... TACATCCTTAATTTAACTCTTCGTCAGTTTCTTTTTTTACTTAAGGAGGACGAATCCATGAAC
PT2-16F AACCAGTTGCTGACTCAGTTGGATGGAGTAGAAGGCTTACAGGGTGTTTATGT ... ---------------------------------------------GAGGACGAATCCATGAAC
PT2-16R AACCAGTTGCTGACTCAGTTGGATGGAGTAGAAGGCTTACAGGGTGTTTATGT ...-- -------------------------------------------GAGGACGAATCCATGAAC
Exon 18: absence of c.2916 del A variant
Intron 22
c.3637-19A>G
Exon 23
c.3637-19A>G variant heterozygous in genomic DNA

## Slide 11
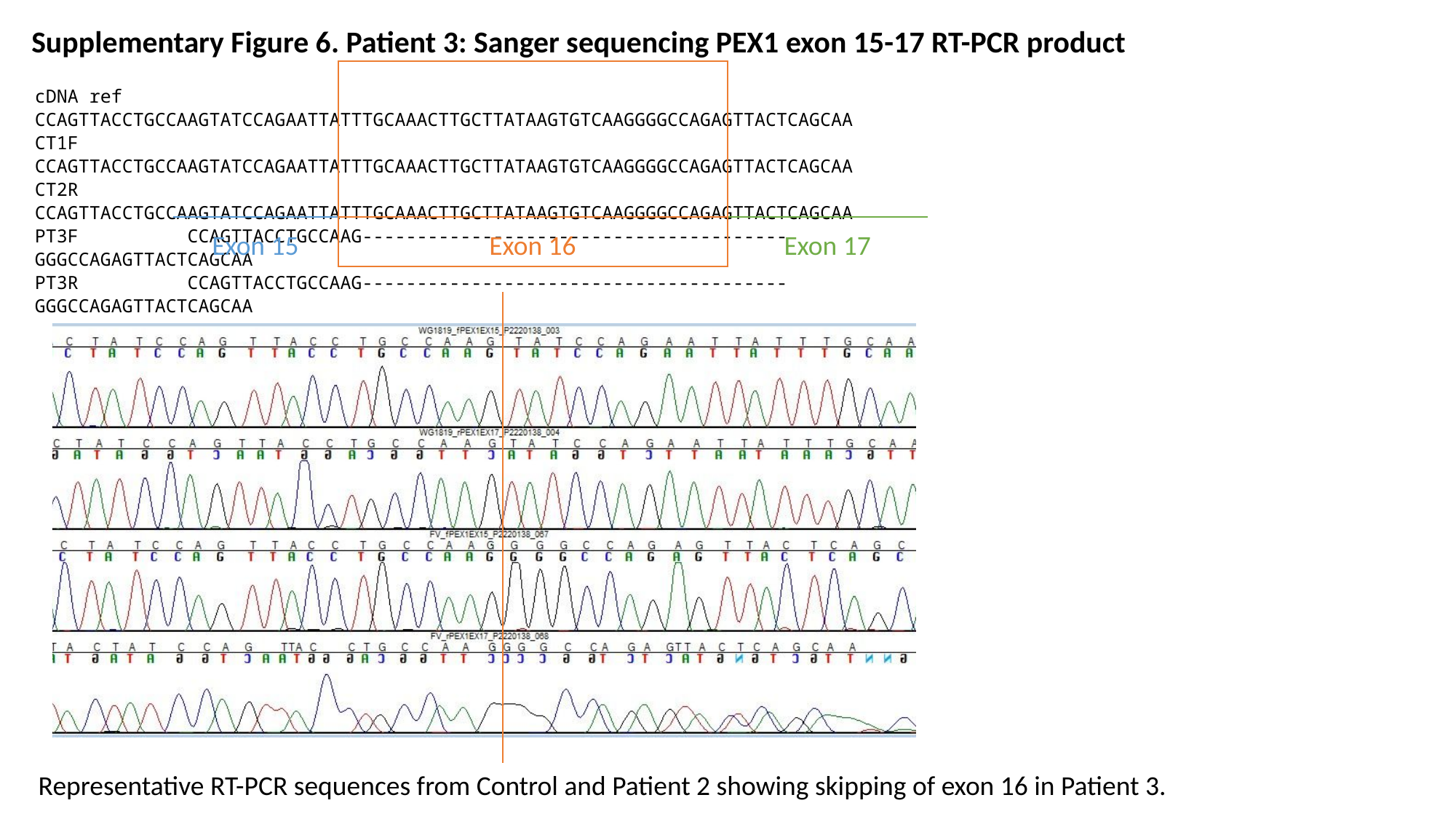

Supplementary Figure 6. Patient 3: Sanger sequencing PEX1 exon 15-17 RT-PCR product
cDNA ref CCAGTTACCTGCCAAGTATCCAGAATTATTTGCAAACTTGCTTATAAGTGTCAAGGGGCCAGAGTTACTCAGCAA
CT1F CCAGTTACCTGCCAAGTATCCAGAATTATTTGCAAACTTGCTTATAAGTGTCAAGGGGCCAGAGTTACTCAGCAA
CT2R CCAGTTACCTGCCAAGTATCCAGAATTATTTGCAAACTTGCTTATAAGTGTCAAGGGGCCAGAGTTACTCAGCAA
PT3F CCAGTTACCTGCCAAG---------------------------------------GGGCCAGAGTTACTCAGCAA
PT3R CCAGTTACCTGCCAAG---------------------------------------GGGCCAGAGTTACTCAGCAA
Exon 16
Exon 17
Exon 15
Representative RT-PCR sequences from Control and Patient 2 showing skipping of exon 16 in Patient 3.
